# *Legionella* relative abundance in shower hose biofilms is associated with specific microbiome members

**DOI:** 10.1093/femsmc/xtad016

**Published:** 2023-08-16

**Authors:** Alessio Cavallaro, William J Rhoads, Émile Sylvestre, Thierry Marti, Jean-Claude Walser, Frederik Hammes

**Affiliations:** Department of Environmental Microbiology, Eawag, Swiss Federal Institute of Aquatic Science and Technology, 8600 Dübendorf, Switzerland; Department of Environmental Systems Science, Institute of Biogeochemistry and Pollutant Dynamics, ETH Zurich, 8092 Zürich, Switzerland; Department of Environmental Microbiology, Eawag, Swiss Federal Institute of Aquatic Science and Technology, 8600 Dübendorf, Switzerland; Department of Environmental Microbiology, Eawag, Swiss Federal Institute of Aquatic Science and Technology, 8600 Dübendorf, Switzerland; Department of Environmental Microbiology, Eawag, Swiss Federal Institute of Aquatic Science and Technology, 8600 Dübendorf, Switzerland; Department of Environmental Systems Science, Institute of Biogeochemistry and Pollutant Dynamics, ETH Zurich, 8092 Zürich, Switzerland; Department of Environmental Systems Science, Institute of Biogeochemistry and Pollutant Dynamics, ETH Zurich, 8092 Zürich, Switzerland; Department of Environmental Systems Science, Genetic Diversity Centre (GDC), ETH Zurich, 8092 Zürich, Switzerland; Department of Environmental Microbiology, Eawag, Swiss Federal Institute of Aquatic Science and Technology, 8600 Dübendorf, Switzerland

**Keywords:** *Legionella*, biofilm, drinking-water-microbiome, correlations pathogen–host interaction

## Abstract

*Legionella* are natural inhabitants of building plumbing biofilms, where interactions with other microorganisms influence their survival, proliferation, and death. Here, we investigated the associations of *Legionella* with bacterial and eukaryotic microbiomes in biofilm samples extracted from 85 shower hoses of a multiunit residential building. *Legionella* spp. relative abundance in the biofilms ranged between 0–7.8%, of which only 0–0.46% was *L. pneumophila*. Our data suggest that some microbiome members were associated with high (e.g. *Chthonomonas, Vrihiamoeba*) or low (e.g. *Aquabacterium, Vannella*) *Legionella* relative abundance. The correlations of the different *Legionella* variants (30 Zero-Radius OTUs detected) showed distinct patterns, suggesting separate ecological niches occupied by different *Legionella* species. This study provides insights into the ecology of *Legionella* with respect to: (i) the colonization of a high number of real shower hoses biofilm samples; (ii) the ecological meaning of associations between *Legionella* and co-occurring bacterial/eukaryotic organisms; (iii) critical points and future directions of microbial-interaction-based-ecological-investigations.

## Teaser

Specific bacteria and eukaryotes are associated with the presence of *Legionella* spp. and *Legionella pneumophila* in shower hose biofilms.

## Introduction

The genus *Legionella* comprises over 60 species, of which about half are recognised opportunistic pathogens and the etiological agents of Legionellosis. *Legionella pneumophila* was the first species identified, and is still linked with most reported Legionellosis cases (Fields et al. 2002). However, multiple other *Legionella* species are ecologically relevant, and sometimes associated with different clinical manifestations of the disease (Muder and Victor 2002, Chambers et al. 2021). *Legionella* are ubiquitous in freshwater, and commonly detected in engineered aquatic ecosystems such as drinking water systems (Falkinham et al. 2015). As such, *Legionella* have been detected in all segments of building plumbing systems, including shower hoses, which often represent a favourable environment for their proliferation and transmission (Falkinham et al. 2015, Proctor et al. 2016, Gebert et al. 2018). In these environments, *Legionella* mainly establish in biofilms, where interactions with other organisms influence their survival and proliferation (Declerck 2010). Shower hoses have been previously linked to the transmission of *Legionella* through inhalation of water aerosol containing the bacteria (Niculita-Hirzel et al. 2022), and ranked second in a relative ranking of *Legionella* exposure pathways from common household water uses (Hines et al. 2014). In a few cases, showers have been directly linked to Legionellosis transmission, specifically in nursing homes, hospitals and hotels (Tobin et al. 1980, Mühlenberg 1993, Bauer et al. 2008), although limited information regarding showers as route of transmission is overall available.

Interactions with their surrounding microbiota may be either favourable or detrimental for *Legionella* (Taylor et al. 2009, Cavallaro et al. 2022). For example, *Legionella* are able to infect a variety of eukaryotes such as amoebae, excavates, and ciliates, escaping their degradation mechanisms and exploiting their intracellular environment for protection and replication (Boamah et al. 2017, Mondino et al. 2020). However, there are also protists such as *Willaertia magna* and *Paramecium multimicronucleatum* that have been documented to degrade *Legionella* under specific environmental conditions (Dey et al. 2009, Amaro et al. 2015, Boamah et al. 2017). Both positive and negative interactions between *Legionella* and other bacteria have also been described previously. For example, *Legionella* was demonstrated to grow in association with Cyanobacteria, as well as with *Flavobacterium breve* (Tison et al. 1980, Berendt 1981, Wadowsky and Yee 1983). Toze and colleagues (Toze et al. 1990) observed that heterotrophic bacteria isolated from chlorinated drinking water were able to support the growth of some species of *Legionella* but inhibited others. Others have also observed inhibition zones surrounding heterotrophic bacteria colonies isolated from various water sources when co-cultured with *Legionella* (Guerrieri et al. 2008, Corre et al. 2018). A few recent studies investigated correlations between *Legionella* and other microbiome members identified through amplicon sequencing. Paranjape and colleagues (Paranjape et al. 2020, Paranjape et al. 2020) conducted two studies on cooling tower water and found that the genus *Pseudomonas* exhibited a strong negative correlation with *Legionella*, which, in turn, positively correlated with the genus *Brevundimonas*; the latter positive interaction has been further demonstrated in laboratory experiments with pure culture isolates. Scaturro and co-workers (Scaturro et al. 2022) investigated building plumbing water samples from several European cities and found positive correlations with bacteria of the genus *Nitrospira*, while observing negative associations with the genera *Pseudomonas* and *Sphingopyxis*.

Despite these correlation or inhibition observations, it remains unclear to which degree results are general or site-specific, whether amplicon sequencing provides sufficient resolution for such complex ecological interactions, and whether a mechanistic understanding of why some members support and other inhibit *Legionella* can be deduced from statistical correlations in microbiome data. Moreover, these studies also focussed exclusively on water samples (with noticeable exceptions, e.g. (Paniagua et al. 2020)), which may not accurately reflect biofilm ecology (Ji et al. 2017), and large sample numbers are sometimes difficult to obtain in such studies.

Here, we extracted 85 shower hose biofilms collected in a multiunit residential building with known *Legionella* contamination that shared the same incoming water, had a similar age, and were made from the same material. We used digital droplet polymerase chain reaction (ddPCR) and amplicon sequencing to identify and quantify potential associations between *Legionella*, bacteria, and eukaryotes in biofilm samples with similar environmental exposure.

## Materials and methods

### Sample collection

Samples were collected from a retirement facility in Switzerland. In total, 85 shower hoses were replaced and transported to the laboratory in sealed bags. All showers-hoses were (i) from the same manufacturer, (ii) of similar operational age, and (iii) fed from the same building plumbing system. Unfortunately, no specific information on use habits (e.g. frequency, temperature, etc.) could be obtained due to privacy protection concerns.

### Biofilm and DNA extraction

Biofilm extraction was performed as described previously (Proctor et al. 2018). Briefly, shower hoses were cut at both ends and filled with sterile glass beads (2 mm diameter) and filtered (0.2 µm) tap water. With both ends plugged, shower hoses were inverted five times in order to allow the beads to distribute internally. Then the hoses were placed in an ultrasonic bath (5 min). After sonication, the shower hoses were unplugged and the biofilm-containing-water was collected in autoclaved Schott bottles. The entire cycle was repeated 5 times, alternating from which end the water was collected. Biofilm-containing-water samples were filtered concentrated (0.2 µm polycarbonate membrane filters) and stored at −20°C until DNA extraction. DNA extraction was carried out using an adapted version of the DNA PowerWater Kit (Qiagen), described by Vosloo and colleagues (Vosloo et al. 2019). Briefly, the filters were fragmented with a scalpel, inserted into 2 mL tubes and treated (i) enzymatically with the addition of Lysozyme; (ii) chemically with the addition of Proteinase K; (iii) mechanically using a tube shaker and the beads provided with the kit, after the addition of chloroform/isoamyl alcohol 24:1. The rest of the extraction was conducted according to the manual provided by the manufacturer of the kit.

### ddPCR for *Legionella* spp. and *Legionella pneumophila*

*Legionella* spp. (ssrA gene) and *L. pneumophila* (mip gene) were measured using a digital droplet polymerase chain reaction (ddPCR) duplex assay with TaqMan chemistry. Gene target primers and probes were based on previously published assays (Benitez and Winchell 2013, Rhoads et al. 2022). Each 25 µL reaction contained 1XPerfeCT a Multiplex ToughMix 5X (Quantabio), 0.6 µM of ssrA and 0.4 µM of mip gene forward and reverse primers, 0.15 µM of each probe, 100 nM Fluorescein (Sigma Aldrich), and 5 µL of DNA template. Primer and probe sequences, fluorophores utilized, master mix composition, and reaction conditions can be found in Table S1. A ddPCR reaction negative control (DNAse free water) was included for each batch of master mix. A ddPCR reaction positive control (Centre National de Référence des Légionelles) was included in each thermocycling run. Droplet formation and PCR thermocycling were performed using Stilla geodes and read using a Prism6 analyser with Crystal Reader software imaging settings pre-set and optimized for PerfeCTa multiplex master mix. Droplets were analysed using Crystal Miner software. Only wells with a sufficient number of total and analysable droplets, as well as a limited number of saturated signals, were accepted according to the Crystal Miner software quality control.

### ddPCR for 16S quantification

Total quantification of the 16S rRNA gene was carried out using a ddPCR assay with an intercalating-dye chemistry. Each 25 µL reaction contained 1X PerfeCTa Multiplex ToughMix 5X (Quantabio), 1.5X EvaGreen (Biotium), 0.8 ng/µL AlexaFluor 488, 0.1 µM of each primer and 5 µL of the DNA template. A ddPCR reaction negative control (DNAse free water) was included for each batch of master mix prepared. A ddPCR reaction positive control was included in each run. The positive control corresponded to the 16S rRNA gene sequence (V4 region) of *Escherichia coli* and was synthesized in the form of gBlock by Integrated DNA technologies (IDT). Primer sequence, master mix composition, and reaction conditions can be found in Table S2.

### Amplification of 16S and 18S rRNA genes for NGS library preparation and illumina sequencing

For sequencing, the V4 region of the 16S rRNA gene and the V9 region of the 18S rRNA gene were amplified by PCR using the primers Bakt_515F—Bakt_805R (Caporaso et al. 2011) and EUK1391F—EUK1510R (Tsao et al. 2019) and the DNA was quantified by Qubit dsDNA HS Assay. Samples were diluted according to their concentration such that 0.1 to 10 ng DNA was loaded into each reaction. Two—step PCR protocol was used to prepare the sequencing library: a first amplification (target PCR) was carried out with 1X KAPA HiFi HotStart DNA polymerase (Roche), 0.3 µM of each 16S primer and 5 µL of 0.1 to 10 ng of template DNA. After amplification, the PCR products were purified with the Agencourt AMPure System (Beckman Coulter, Inc.). The second PCR (adaptor PCR) was performed with limited cycles to attach specific sequencing Nextera v2 Index adapter (Illumina). After purification, the products were quantified and checked for correct length (bp) with the High Sensitivity D1000 ScreenTape system (Agilent 2200 TapeStation). Sample concentration was adjusted and samples were subsequently pooled together in a library at a concentration 4 nM. The Illumina MiSeq platform was used for pair-end 600 cycle (16S) and paired-end 300 cycle sequencing with 10% PhiX (internal standard) in the sequencing run. Negative controls (PCR grade water) and a positive control (For 16S library; self-made MOCK community) were incorporated. Primer sequences, master mix composition, and reaction conditions can be found in the Table S3. Experiments and data on community composition were generated in collaboration with the Genetic Diversity Centre (GDC) of ETH Zurich.

### Data analysis

16S and 18S rRNA sequencing data were processed on HPC Euler (ETHZ) using workflows established by the GDC, ETHZ. Detailed data processing workflows are provided in the supplementary materials. For the 16S dataset, because of the low quality of the reverse read, only the forward read has been processed further and used for the rest of the analysis. Since the primers amplified only the V4 region of the 16S, excluding the reverse read from the analysis did not cause a loss in length of the amplicon. For the 16S dataset, all R1 reads were trimmed (based on the error estimates) by 25 nt, the primer region removed, and quality filtered. For the 18S dataset, all read pairs were merged, primer sites removed, and quality filtered. Ultimately, using UNOISE3 (Edgar and Flyvbjerg 2015), sequences were denoised with error correction and chimera removal and Amplicon Sequence Variants (ASV) established. In this study, the predicted biological sequences will be referred to as Zero-Radius Operational Taxonomic Units (ZOTUs). Taxonomic assignment was performed using the Silva 16S database (v128, 16S amplicon sequencing) and PR^2^ (V4.12.0, 18S amplicon sequencing) in combination with the SINTAX classifier. Samples were not rarified to avoid the loss of data due to differences in the sequencing depth (McMurdie and Holmes 2014). Alpha-diversity (calculated with Shannon Index, which measures intra-sample diversity accounting for both richness and evenness), distance ordination and average relative abundance analysis were performed using R (version 4.2.1) and R studio (version 2022.07.2+576) using the packages ‘ggplot2’, ‘microbiomeExplorer’ (through a Shiny app, (Reeder et al. 2021)) and the Bioconductor ‘phyloseq’ (version 1.42.0, (McMurdie and Holmes 2013)). SparCC correlation analysis was performed with the R package ‘SpiecEasi’ and built as a network with Cytoscape (version 3.9.1). Unless otherwise specified, all packages were operated using the default settings. Random Forest analysis was performed using Microbiome Analyst (Chong et al. 2020). Briefly, the samples were filtered based on a low count filter (minimum count of 4 reads with 20% prevalence in samples) and a low variance filter (10% removal with the variance measured using Interquartile-range), using the Microbiome Analyst default settings. Then, the experimental variable was set (*Legionella* spp. or *L. pneumophila* relative abundance) and a Random Forest is run with 1000 trees, 7 predictors and a Randomness setting option. The contribution of each predictor to the model was calculated and reported as Mean Decrease Accuracy (MDA), which indicates how much the model loses confidence if that predictor is removed from the model itself. The Out Of Bag (OOB) error was used as a validation parameter for the accuracy of the models. In general, the OOB value is a result of 1–accuracy. Furthermore, analyses were performed at genus level as well as at ZOTU level in order to detect associations that would not be displayed when grouping single sequences into large genus categories. All the remaining graphs were constructed with the R package ‘ggplot2’ (version 3.4.0).

## Results

### Microbial ecology of shower hoses biofilms

**Figure 1.**
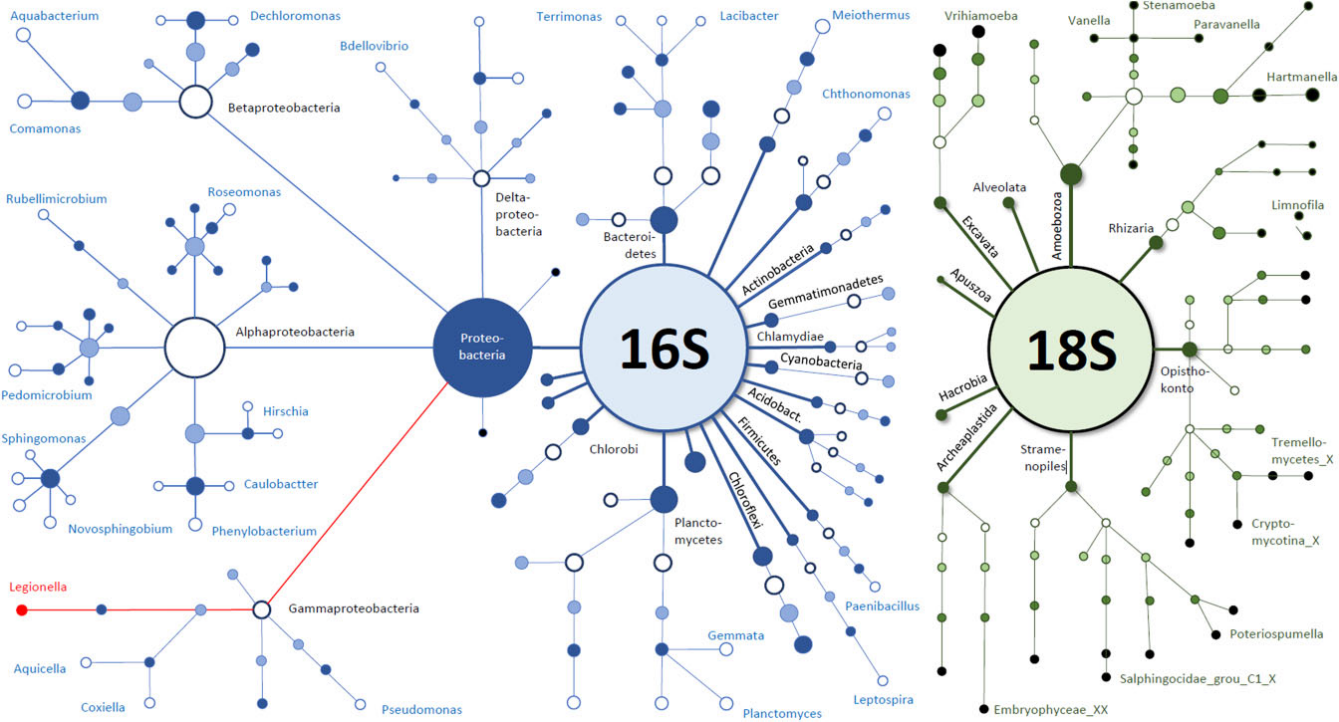
Overview of the average microbial composition of the 85 shower hose biofilms collected from a single building for this study. The figure shows average 16S and 18S data (n = 85) for ZOTUs above 0.01% relative abundance. Size of the nodes depicts the relative abundance of the corresponding taxa, while the connectors show the connection to the lower taxonomic level (from phylum to genus). Nodes are shown until the last level that is taxonomically assigned. Unclassified taxa (not shown) at genus level account for 64.4±1.4% of the bacterial community and 85.3±1.2% of the eukaryotic community.

Shower hose biofilms fed with non-chlorinated drinking water consist of complex and diverse prokaryotic and eukaryotic communities (Fig. 1). Amplicon sequencing revealed 1518 ZOTUs (16S, bacterial) and 1389 ZOTUs (18S, eukaryotic), respectively, with 111 bacterial and 78 eukaryotic taxa classified at genus level. These numbers represent a gross underestimation of the true diversity, since unclassified taxa at genus level accounted on average for 64.4±1.4% of the bacterial community and 85.3±1.2% of the eukaryotic community. Among the bacterial genera, *Meiothermus* spp. (3.6±0.6%), *H16* spp. (2.5±0.2%) and *Caulobacter* spp. (3.2±0.6%) were the most abundant, with the top ten organisms representing 19.6% of the identified community at the genus level. The eukaryotic community was composed of 10 phyla, of which *Amoebozoa* (7.8±0.9%)*, Excavata* (3.2±0.4%) and *Opisthokonta* (4.0±0.6%) were most abundant. Among the eukaryotic community, an average of 78±1.3% of the taxa were unclassified even at phylum level (in contrast, 0% of the bacterial community was unclassified taxa at phylum level). At the genus level, the three most abundant known eukaryotic taxa were *Hartmannella* (4.5±0.9%), *Limnofilila* spp. (1.6±0.3%) and *LKM74* (1.6±0.2%).

**Figure 2.**
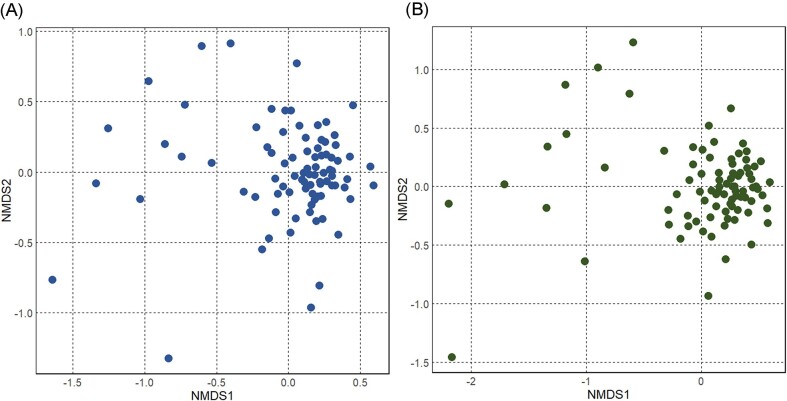
Ordination analysis of the **(A)** 16S and **(B)** 18S rRNA amplicon sequencing data from 85 shower hose biofilm samples, stress = 0.189 The graphs show distance among samples, calculated using the Bray–Curtis method and plotted on NMDS graphs.

The distance between samples was measured using the Bray-Curtis index for both 16S and 18S data (Fig. 2). In both cases, the samples did not show significant clustering with respect to the limited metadata available for this study (such as the location in the building where the sample campaign took place), indicating that the overall microbial composition of the samples was similar, with a few notable exceptions. However, relative abundance of individual taxa at genus level shows noticeable variations among samples (Fig. S1). In both 16S and 18S data, 12 samples (four shared; S001, S004, S008, S101) clustered separately from the bulk of the samples. This was likely due to the low richness of these samples. Alpha-diversity was measured using the Shannon index (H), which evaluates both richness and evenness of the species present. The analysis for the 16S data set shows high H values within samples, ranging between 3 and 5, with 11 samples having less than 3. The same analysis performed on the 18S dataset shows that most samples range between 3 and 4.8, while 12 samples have values below 3 (‘H’, Fig. S2).

### *Legionella* spp. and *L. pneumophila* have different relative abundances across samples

The genus *Legionella* was a predominant taxon in many samples. With an average relative abundance of 0.27±0.02%, it ranked among the 30 most abundant genera in the community. Notably, 30 unique ZOTUs associated with the genus *Legionella* (the highest number of ZOTUs for a genus in this dataset) were detected and the relative abundance of these varied considerably across samples from the same building (Fig. S3).

**Figure 3.**
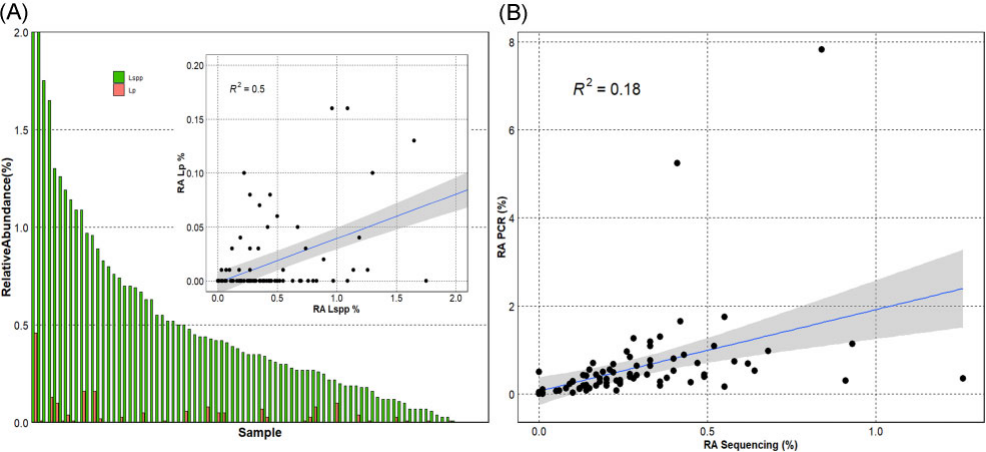
**(A)** Relative abundance of *Legionella* spp. (green bars) and Legionella pneumophila (orange bars) measured with ddPCR. Absolute quantification of *Legionella* spp. and *L. pneumophila* was measured with a duplex ddPCR assay, and concentrations were then normalized relative to the total 16S rRNA gene concentrations. The inlay figure displays the linear correlation (r = 0.5) between the relative abundance of *Legionella* spp. and *L. pneumophila* among samples (RA=Relative Abundance); **(B)** Linear correlation plot of the relative abundance of *Legionella* spp. calculated both with ddPCR and sequencing data.

We performed ddPCR for specific quantification of *Legionella* spp. and *L. pneumophila*, as well as for quantification of total 16S rRNA gene abundance (Fig. 3). Subsequently, the abundance of *Legionella* spp. and *L. pneumophila* was calculated relative to the total 16S rRNA abundance, and this relative abundance was compared across samples. *Legionella* spp. (relative abundance: 0.001% to 7.8%) was detected in all samples, while *L. pneumophila* (relative abundance: 0 to 0.46%) was detected in 34% of samples (Fig. 3A). No clear correlation was observed between the relative abundance of *Legionella* spp. and *L. pneumophila*. Importantly, the relative abundance of *Legionella* spp. calculated from the 16S rRNA amplicon sequencing data and that calculated with ddPCR *(Legionella* spp. and *L. pneumophila* relative to the total 16S rRNA) correlated poorly (R^2^=0.18; Fig. 3B). Unless stated otherwise, the following analysis uses the targeted ddPCR data.

This study focused on the interactions between microbiome members and the absolute abundance of *Legionella* in the hoses were not quantified. However, based on the ddPCR data and the average biofilm volumes sampled, *Legionella* spp. were present at values between approximately 1E+3–1E+7 GU/hose., which is in agreement with previous studies (Proctor et al. 2018). *Legionella pneumophila* were considerably less abundant at approximate values ranging from 0 to 1E+06 GU/hose.

### Associations between *Legionella* spp. and members of the community

**Figure 4.**
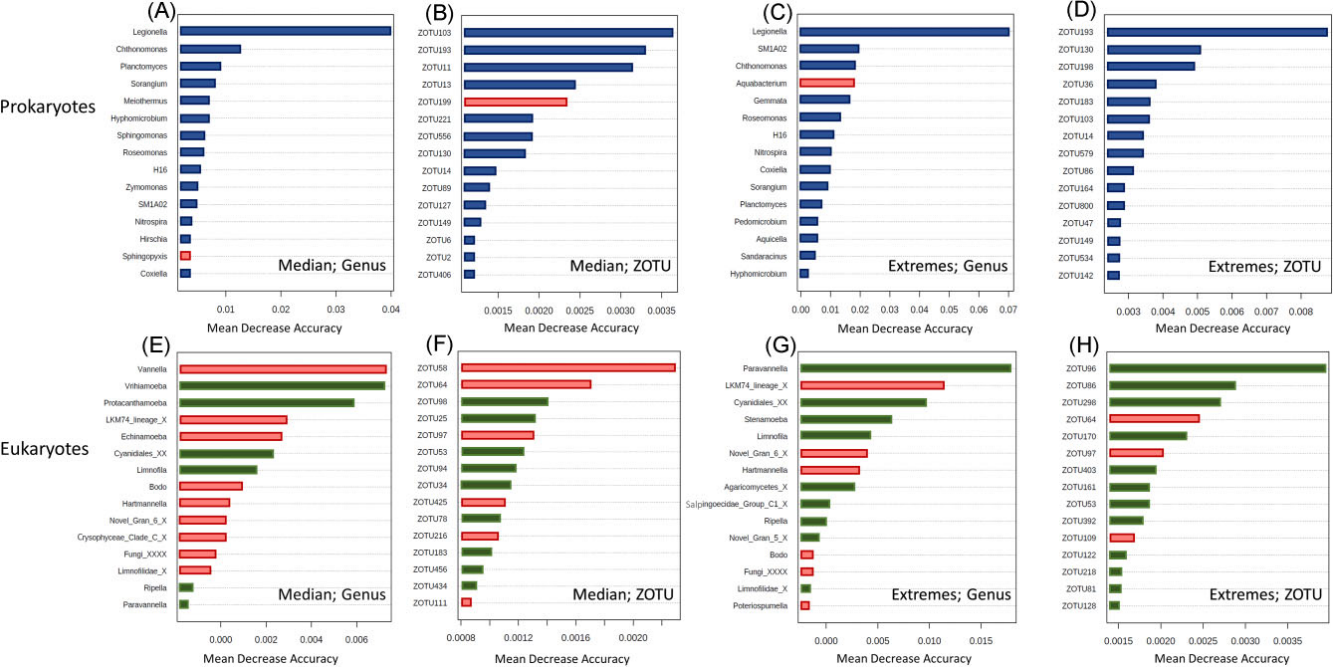
Random Forest analysis of the 16S and 18S datasets using the relative abundance of *Legionella* spp. as variable. Red represents predictors of low *Legionella* abundance while blue and green represent predictors associated with high abundance for bacteria and eukaryotes, respectively. **A-B**: Main bacteria (genus level, A; ZOTUs level, B) predicting *Legionella* spp. abundance with a threshold of 0.3%; **C-D**: Main bacteria (genus level, C; ZOTU level, D) predicting *Legionella* spp. abundance using the samples at the extremes. **E-F**: Main eukaryotes (genus level, E; ZOTU, F) predicting *Legionella* spp. abundance using 0.3% as threshold; **G-H**: Main eukaryotes (genus level, G; ZOTU, H) predicting *Legionella* spp. abundance using the samples at the extremes.

Random forest analysis identified combinations of bacterial and eukaryotic taxa that predicted the presence of high and low relative abundance of *Legionella* spp., but the choice of the model threshold affected the prediction (Fig. 4, Figs S5 and S6). Using a median relative abundance threshold of 0.347% (equalling 42 samples with ‘high’ relative abundance and 43 samples with ‘low’ relative abundance), the model (at genus level) showed an OOB error of 0.275 for the 16S dataset (Fig. 4A) and 0.43 for the 18S dataset (Fig. 4E). At the ZOTU level, the model recorded an OOB error of 0.325 for the 16S (Fig. 4B), and 0.376 for the 18S (Fig. 4F) datasets. Main bacterial predictors of high relative abundance of *Legionella* spp. across models included *Chthonomonas, Planctomyces*, and *Sorangium*, with MDAs between 0.01 and 0.015 (Fig. 4A), as well as ZOTU103 (g_*Reyranella*), ZOTU193 (g_*Legionella*), ZOTU11 (g_*Chthonomonas*) and ZOTU13 (g_*H16*) with MDAs between 0.003 and 0.004. Only two predictors of low *Legionella* spp. relative abundance, namely *Sphingopyxis* (MDA close to 0) and ZOTU 199 (MDA 0.0025; g_*Aquabacterium*) were detected. For the 18S dataset (Fig. 4E), more predictors of low *Legionella* spp. abundance were observed (9 out of 15 predictors). The genera *Vanella* (linked to low abundance, MDA 0.008), *Vrihiamoeba* (linked to high abundance, MDA 0.008), and *Protacanthamoeba* (linked to high abundance, MDA 0.006) were the three main classified eukaryotic predictors of *Legionella* spp. relative abundance. ZOTU 58 (unclassified) predicted low *Legionella* abundance with an MDA of 0.025.

However, selection of the criteria, such as the range of relative abundances to analyse for the model, is critical. When performing the analysis only using the samples at the extremes (15 samples with highest relative abundance; 15 samples with lowest relative abundance), the model returned an OOB error of 0.225 (16S) and 0.567 (18S) at the genus level and 0.2 (16S) and 0.5 (18S) at the ZOTU level (Fig. 4A-D). For the bacterial community, *SM1A02* and *Chthonomonas*, consistently with the previous model, predict high *Legionella* abundance with MDAs of 0.0175, as did ZOTU193 (g_*Legionella*, MDA 0.01), ZOTU130 (c_*Acidobacter subgroup*, MDA 0.007) and ZOTU198 (g_*Coxiella*, MDA 0.005). *Aquabacterium* was the only trait that predicted low *Legionella* abundance (MDA 0.15.; Among the eukaryotic predictors, the genera *Paravannella* and *LKM74_lineage_X* consistently with the models described above, predict respectively high and low *Legionella* spp. abundance (MDA 0.0175; MDA 0.012). Three predictors of low *Legionella* spp. abundance are detected at the ZOTU level (ZOTU64; ZOTU97; ZOTU109, unclassified sequences). ZOTU96 (unclassified) was the stronger predictor of high *Legionella* spp. relative abundance, with an MDA of 0.004. We furthermore tested the models decreasing the thresholds between samples with high and low *Legionella* relative abundance to 0.2% (27 samples with low relative abundance; 58 samples with high relative abundance) and 0.1% (17 samples with low relative abundance; 68 samples with high relative abundance), which generated groups with uneven sample distribution and displayed different associations (Figs S5 and S6).

### Associations between *L. pneumophila* and members of the biofilm microbiome

We also identified predictors of *L. pneumophila* relative abundance using Random Forest analysis. Using the median value (0.002%, Fig. 3) as a threshold for samples with high and low relative abundance of *L. pneumophila*, the model performed with an OOB of 0.4 (16S) and 0.529 (18S) at genus level, and with OOB of 0.352 (16S) and 0.435 (18S) at ZOTU level. In contrast to the observations for *Legionella* spp. (above), the analysis showed more predictors of low abundance (eight at genus level; seven at ZOTU level). *Rhodobacter* (MDA of 0.014) and ZOTU57 (c_TK10, MDA 0.017) predicted low *L. pneumophila* relative abundance, while ZOTU52 (g_*Pedomicrobium*; MDA 0.030) was the most important predictor of high relative abundance. Only 10 features were detected with three negative associations, of which *LKM74_lineage_X* resulted as the most important (MDA 0.007). At the ZOTU level for the 18S dataset, the first three predictors are all associated with high *L. pneumophila* numbers (ZOTU342; ZOTU181; ZOTU168, unclassified sequences), while six ZOTUs predicted low *L. pneumophila* abundance, of which ZOTU230 (unclassified) had the highest importance.

We furthermore performed the analysis using only the extreme samples (25 samples highest abundance, 25 samples lowest abundance; Fig. 3). Here, the model performed with an OOB of 0.38 and 0.5 at the genus level, and 0.32 (16S) and 0.46 (18S) at the ZOTU level. For the 16S data, the results were similar to the previously described model, with more predictors of low *L. pneumophila* relative abundance (10). Interestingly, the model was not able to associate any predictors to the abundance of *L. pneumophila* for eukaryotes using these model parameters. At the ZOTU level, the bacterial ZOTU869 (g_*Legionella*) had the strongest importance value and an association with high *L. pneumophila* abundance. In general, more bacterial ZOTUs (11) predicted high *L. pneumophila* abundance in this model. For the eukaryotic sequences, the first three important ZOTUs predict low *L. pneumophila* abundance (ZOTU134; ZOTU48; ZOTU64, unclassified sequences).

### *Legionella* ZOTUs correlations in a SparCC network analysis

SparCC analysis revealed several correlations between specific *Legionella* ZOTUs and other taxa, using only the 16S amplicon sequencing data. Figure 6 shows only significant correlation coefficients with a threshold of ±0.3, with a minimum of −0.36 and a maximum of 0.63. Since not all ZOTUs are assigned to the same level of taxonomy, the lowest level available was used to describe our observations. Of the 30 sequences associated to the *Legionella* genus, 18 ZOTUs did not display any significant correlation with other microbiome members above the threshold. The remaining 12 ZOTUs correlated in different ways with other sequences. For example, ZOTU193 was the most prevalent sequence assigned to the genus *Legionella* in the dataset (Fig. S3) and was also the one displaying the most correlations (67), the majority of which were positive. Only four negative correlations have been detected, with ZOTU104 (g_*Ferribacterium*), ZOTU765 (f_*Obscuribacterales*), ZOTU156 (g_*Lacibacter*), and ZOTU1309 (g_*Rhodoplanes*). The strongest positive correlation (0.63 correlation coefficient) is with ZOTU33 (g_*H16)*.

The analysis also showed that *Legionella* ZOTUs associated in different ways with the same taxa. For example, while the *Legionella* ZOTU193 positively correlated with ZOTU135 (o_*Obscuribacterales*) and ZOTU196 (f_*Rhodobiaceae*) while *Legionella* ZOTU1258 displayed negative correlations with these. The same phenomenon was observed for ZOTU55 (g_*SM1A02*, positively correlates with *Legionella* ZOTU193, negatively correlates with *Legionella* ZOTU1483) and for ZOTUs 323 and 37, (f_*Planctomycetaceae*; g_*Pedomicrobium*; positively correlate with *Legionella* ZOTU193 and negatively correlate with *Legionella* ZOTU1379). ZOTU996 mostly displayed negative correlations (nine, ranging from −0.3 to −3.6): the only positive correlation (coefficient of 0.322) was with ZOTU817 (g_*Bdellovibrio*), while ZOTU78 (g_*Nitrospira*), ZOTU347 (g_*H16*) were the ones with the highest negative correlation coefficients. In only two cases, two *Legionella* ZOTUs correlated with each other, in both cases positively: ZOTU735 and ZOTU640 correlated with each other (correlation coefficient 0.3), as well as ZOTU462 and ZOTU241 (correlation coefficient 0.31). The genus *Chthonomonas was always* associated with high *Legionella* spp. abundance and positively correlated with the *Legionella* ZOTU801 (correlation coefficient 0.3).

## Discussion

**Figure 5.**
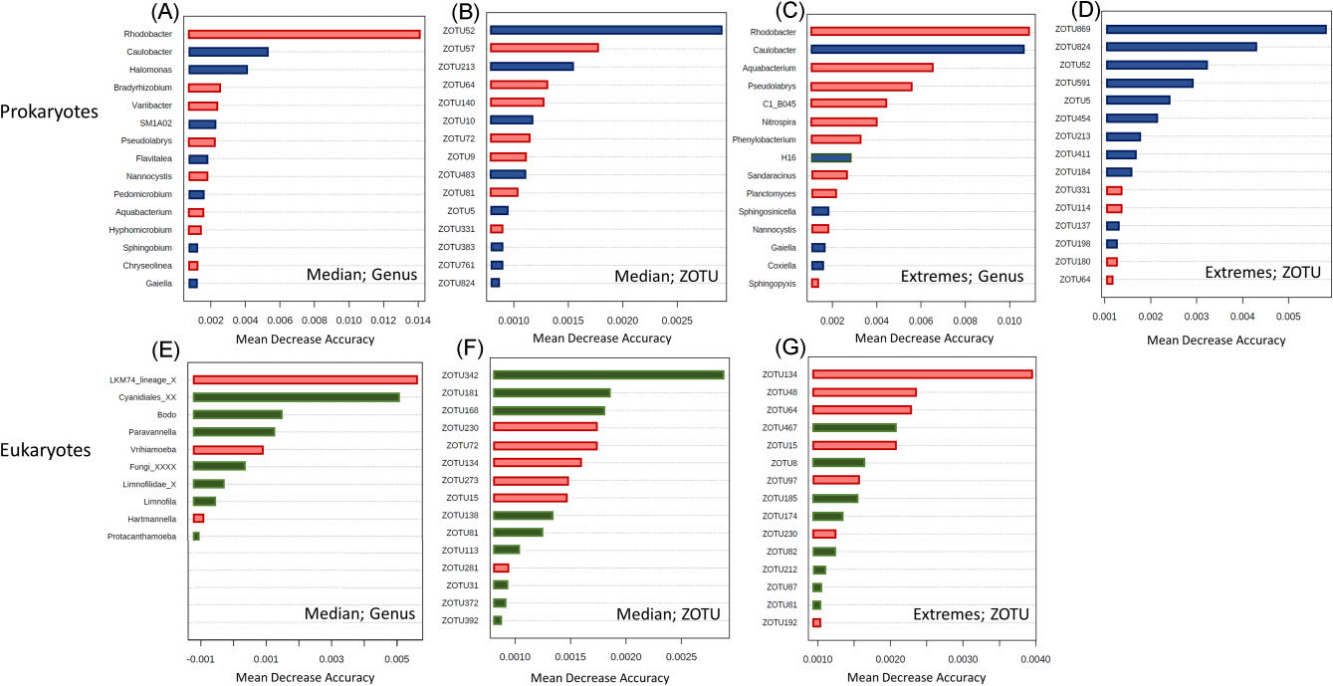
Random Forest analysis of 16S and 18S data sets using the relative abundance of *Legionella pneumophila* as variable. Red represents predictors of low *Legionella* abundance while blue and green represent predictors associated with high abundance for bacteria and eukaryotes respectively. **A-B**: Main bacteria (genus level, A; ZOTUs level, B) predicting *L. pneumophila* abundance with a threshold of 0.02% relative abundance; **C-D**: Main bacteria (genus level, C; ZOTU level, D) predicting *L. pneumophila* abundance using the samples at the extremes. **E-F**: Main eukaryotes (genus level, E; ZOTU, F) predicting *L. pneumophila* abundance using 0.02% relative abundance as threshold; **G**: Main eukaryotes (ZOTU, G) predicting *L. pneumophila* abundance using the samples at the extremes.

**Figure 6.**
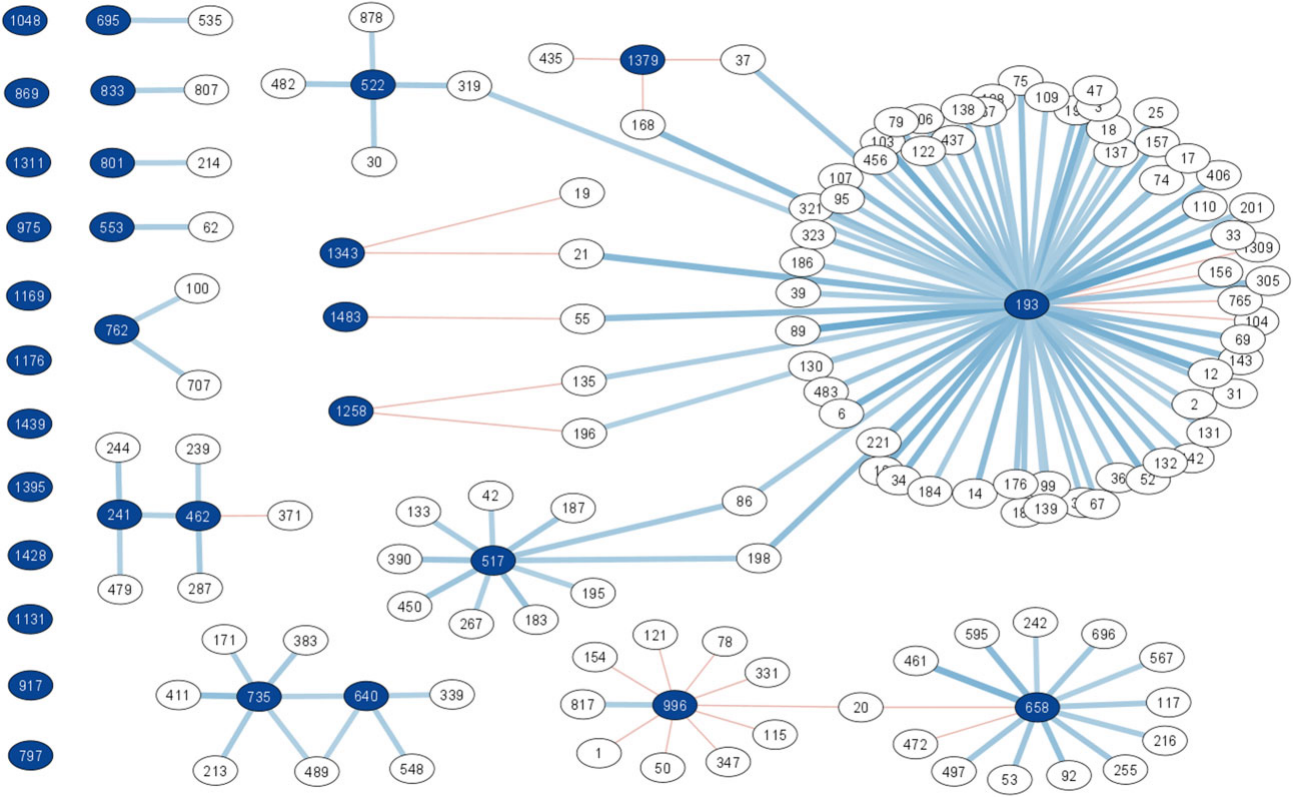
SparCC analysis constructed as a network using the 16S amplicon sequencing dataset, subsetted for the ZOTUs associated with *Legionella* taxonomy. To help interpretation, the nodes corresponding to the *Legionella* ZOTUs are coloured in dark blue, while the edges are in light blue indicating positive correlations and orange for negative correlation coefficients. In the far left are all the *Legionella* ZOTUs that do not show any significant correlation (above 0.3 correlation coefficient).

Community analysis of 85 shower hose samples revealed a highly rich bacterial and eukaryotic composition (Fig. 1) that was relatively consistent among samples (Fig. 2). However, *Legionella* spp. and *L. pneumophila* relative abundance varied considerably among samples (Fig. 3). Various Random Forest analyses demonstrated that several bacterial and eukaryotic taxa were associated with either high or low relative abundance of *Legionella* spp. and respectively *L. pneumophila* (Figs 4-5). Moreover, SparCC correlation analysis showed how individual *Legionella* ZOTUs correlated with specific microbiome members (Fig. 6).

### Microbial ecology in the final meters of water distribution

Shower hoses biofilms are inhabited by diverse prokaryotic and eukaryotic communities. Three main factors promote microbial growth here: (i) temperature, which is often warmer in the last meters; (ii) flexible materials that leach organic carbon; (iii) usage patterns that often create extensive stagnation periods (Proctor et al. 2016). This makes shower hoses ideal environmental niches where biofilms form and microorganisms can proliferate. Differences in the above-mentioned factors also means that there is considerable diversity between different shower biofilm microbiomes (Proctor et al. 2016, Gebert et al. 2018). To mitigate the uncertainty caused by such differences, we collected all hoses on purpose from a single building, which shared the same incoming water, had a similar age, and were made from the same material. The shower hose biofilms showed general similarity in terms of common diversity indices, with dominant taxa similar to those identified from previous studies of non-chlorinated drinking water systems (Liu et al. 2014, Proctor et al. 2018, Neu et al. 2019). The average relative abundance of the microorganisms is highly variable among biofilm samples (Fig. S1), as well as the relative abundance of *Legionella* spp. and *L. pneumophila*, and *Legionella*-associated ZOTUs (Fig. 3, Fig. S3). While the reasons behind this variability are not clear, the data provided interesting information: (i) *Legionella* spp. relative abundance reaches very high values in shower hoses biofilms (7.8% in our samples); (ii) the higher relative abundance of *Legionella* spp. compared to *L. pneumophila*, as well as the 30 ZOTUs classified as *Legionella*, suggest multiple *Legionella* species inhabiting individual shower hose biofilms. Despite the lack of available studies, the presence of multiple *Legionella* species with different abundances has been detected in different water systems, although poorly described in shower hoses to our knowledge (Lesnik et al. 2016, Pereira et al. 2017, Logan-Jackson and Rose 2021, Salinas et al. 2021, Gleason et al. 2023).

Many studies cover prokaryotic drinking water biofilms, but information on eukaryotes in these systems is still severely limited. Studies focussing on eukaryotes detected amongst others the fungal sub-phylum *LKM11* and the amoeba clade *LKM74* in biofilm samples of different DWDSs, together with other organisms like *Streptophyta*, Ciliates and Algae, *Opisthokonta, Stramenopiles and Alveolata* (Inkinen et al. 2019, Soler et al. 2021). We also detected these taxa, but, importantly, as well as several taxa that are known carriers of opportunistic pathogens, such as *Vannella* spp., *Hartmannella* spp., *Acanthamoeba* spp. and *Echinamoeba* spp. (Inkinen et al. 2019). However, the relevant eukaryotic community is likely under-represented with current 18S amplicon sequencing tools. For example, 18S primers target all eukaryotes, which increases the chances of detection of undesired species/contaminations. In response, specific databases have been developed for certain fractions of eukaryotes of interest to the exclusion of others (Guillou et al. 2013). A further major drawback is that the taxonomic identification of eukaryotes is still limited and this calls for improvements in all aspects of eukaryotes ecology research, from targeted sample collection to dedicated analysis pipelines (Gabrielli et al. 2023).

All this information together highlight how shower hoses biofilms are rich environments, where interactions happen from species to kingdom level (Sadiq et al. 2022). As shown, *Legionella* spp. are members of these environments, and therefore subjected to different ecological interactions.

### Some bacterial taxa are associated with *Legionella* abundance

Microbes in biofilms exist in close proximity, thus creating opportunities to establish positive and/or negative interactions with each other. Random Forest analysis showed a prevalence of positive associations between the resident microbiomes and *Legionella* spp./*L. pneumophila* (Figs 4 A–D and 5 A–D). Positive bacterial interactions include promoting the growth of other bacteria by increasing general nutrient availability, creating new niches or directly exchanging important nutrients (e.g. cross feeding of amino acids) (D'Souza et al. 2018, Kehe et al. 2021). In the context of *Legionella* growth, such beneficial interactions can play a decisive role: *Legionella* are auxotrophs for at least seven amino acids (Tesh and Miller 1981, Chien et al. 2004).

In contrast, negative associations between bacteria and *Legionella* can include direct (interference) or indirect (exploitative) competition (Granato et al. 2019, Cavallaro et al. 2022). Our results suggest a negative association between *Legionella* spp. and only two genera, namely *Aquabacterium* and *Sphingopyxis* (Fig. 4). It is not possible to exactly establish the nature of these interactions using only amplicon sequencing data. While these two taxa are not known to be producers of secondary metabolites with antimicrobial activity, they are characterized by a highly versatile metabolism, which provides advantages with respect to surviving to unfavourable environmental conditions (Sharma et al. 2021). This can in theory give them an advantage in terms of competition for resources in low-nutrient environments compared to other microorganisms, especially the ones with specific and high nutrient requirements, like *Legionella*.

A few previous studies have explored associations between *Legionella* and the resident microbiome in different systems. In two studies focusing on cooling towers, Paranjape and colleagues found bacteria associated with the presence or absence of *Legionella* spp. and *L. pneumophila* (Paranjape et al. 2020, Paranjape et al. 2020). *Pseudomonas* was found as the main genus correlating with the absence of *Legionella*, as already reported elsewhere (Leoni et al. 2001, Proctor et al. 2018). Interestingly, our study did not show a strong negative association between *Pseudomonas* and *Legionella*. In addition, Paranjape and co-workers reported *Sphingopyxis* as a positive correlating genus, in contradiction with our results (Fig. 4). A recent investigation on plumbing systems across four cities of Europe revealed associations between culturable *Legionella* and the microbial communities detected in the water samples (Scaturro et al. 2022). While reinforcing the negative association between *Legionella* and *Pseudomonas*, the authors also described a negative association with the genera *Staphylococcus* and, consistently with our analysis, *Sphingopyxis*. Any specific associations between the genera detected in this study and *Legionella* were, to our knowledge, not previously described. Notably, two genera among the ones associated with Legionella (i.e. *Meiothermus* and *Chthonomonas*) are thermophilic (Lee et al. 2011, Ho et al. 2016), suggesting the possibility that some of these associations are dictated by environmental preferences (i.e. shared niches) rather than actual direct interactions. However, Chthonomonas spp. is also known for establishing mutualistic relationship with other organisms, specifically in biofilms (Stott et al. 2018).

Associations between bacterial communities and culturable *L. pneumophila* in hot and cold water were also investigated in a study conducted on high rise buildings in New York City (Ma et al. 2020). In that study, associations were described at higher taxonomic levels and negatively linked *L. pneumophila* and members of the classes *Bacteroidia* and *Solibacterales*, while positive associations were detected with *Chloracidobacteria, Gemm-1*, and *Actinobacteria*, among others. The fact that disagreement exists between the various studies depends on different factors. For example, previous studies analysed exclusively the water phase, in contrast with our study that focused on the biofilm phase. Moreover, the taxonomic classification at the genus (sometimes class) level that was used in all of these studies does not allow for specific identification of the organisms involved, and different species within the same genus might exhibit different associations with the same organism. We explored the latter point by looking at the correlations that specific *Legionella* ZOTUs exhibit in a SparCC analysis (Fig. 6; discussed below). The real ecological meaning of these associations is not clear, as correlation can obviously not be linked automatically to causation. Many microbial ecology studies aim to find associations between two species that reflect their ecology in the environment (Carr et al. 2019). However, interactions between species often occur in high-order combinations, where an interaction between two species is modulated by a third one (Bairey et al. 2016). Therefore, associations detected by statistical analysis may not necessarily describe direct interactions between microorganisms. It is also possible that two organisms are correlating with a common environmental variable rather than each other. Nevertheless, these observational studies create good opportunities to further study bacterial interactions using laboratory approaches. For example, Paranjape et al. (Paranjape et al. 2020) isolated a strain of *Brevundimonas* and confirmed the positive effect of the strain towards the growth of *L. pneumophila* previously observed through statistical analysis.

### Some eukaryotic taxa are also associated with *Legionella* abundance

Random forest analysis performed on the eukaryotic data set revealed more negative associations with *Legionella* abundance compared to the bacterial data. Unravelling the meaning of these associations is challenging, as this is likely linked to the intracellular lifestyle of *Legionella*. Interactions between protists and *Legionella* can, in fact, have very different outcomes. For example, *Legionella* are taken up by their host as a potential food source, but escape the host's degradation mechanisms and start the replicative phase by establishing in the *Legionella*-containing-vacuole (LCV) (Boamah et al. 2017). When this happens, the protist hosts are often killed (thus resulting in increased *Legionella* abundance and decreased host abundance). Alternatively, some protists effectively graze on *Legionella* as a food source and are resistant to their escape mechanisms, resulting in decreased *Legionella* abundance and increased host abundance (Dey et al. 2009, Amaro et al. 2015, Boamah et al. 2017, Mondino et al. 2020). Paranjape and co-workers interpreted a positive association between *Legionella* and *Oligohymenophorea* as an indication of host-prey relationship in which *Legionella* is able to replicate (potentially without causing the death of the host) (Paranjape et al. 2020). To add to the complexity, it has been reported that the temperature and the abundance of *Legionella* can change the fate of host-prey relationships, which can be either digested, packaged into vesicles and secreted in pellets, or survive intracellularly with no replication (Boamah et al. 2017).

Different *Legionella* species can also be associated with different outcomes with the same host (Boamah et al. 2017). Our dataset identified several associations with protists proposed as *Legionella* hosts. *Vannella* spp., *Echinamoeba* spp. and *Hartmannella* spp. (now *Vermamoeba*) predicted the low abundance of *Legionella* in the biofilm samples. While *Vannella* has been previously described as able to kill *Legionella* (Rowbotham 1986), *Vermamoeba* is often referred to as a permissive host for *Legionella*, although cases of intracellular survival have been reported (Buse and Ashbolt 2011). To our knowledge, no information on how the interaction *Echinamoeba*-*Legionella* takes place is available. Additional negative associations include *Novel-Gran-6*, which are members of the phylum *Cercozoa*, described as particularly prone to digest *Legionella (**Boamah et al. 2017*). The genera *Vrihiamoeba* and *Protacanthamoeba* predicted, on the other hand, high *Legionella* abundance. No clear interaction between these taxa and *Legionella* has to our knowledge been described, but both are known to prey on bacteria (Delafont et al. 2014, Ma et al. 2016). Interestingly, *Vrihiamoeba* is a predictor of low *L. pneumophila* abundance, indicating that the same interaction could produce a different outcome depending on the specific *Legionella* species involved.

### Associations are often interactions that are happening at small taxonomic scale

A limitation of community analysis performed through 16S amplicon sequencing is that genus-based generalization of the interactions is usually made, while species dynamics is not considered. In response, we explored the diversity and associations of *Legionella* ZOTUs (Fig. 6; Fig. S3). Previous studies discussed the possibility to identify species of *Legionella* using only a partial sequence of the 16S rRNA gene (Wilson et al. 2007, Ma et al. 2020). If correct, this offers an opportunity to study ecological dynamics of the genus *Legionella* at species level in environmental samples.

While this evidence combined suggest that different species of *Legionella* inhabit building plumbing biofilms, the Random Forest analysis used either the entire genus or specifically *L. pneumophila* datasets as external variables for the predictions. To challenge this further, we focused on the correlations of the individual *Legionella* ZOTUs, which suggested that the 30 sequences display markedly different associations in the complex ecological network represented by these biofilms. A first observation of this network, consistently with the Random Forest data, highlights a prevalence of positive associations between *Legionella* and the resident microbiome. In the specific case of this study, different *Legionella* sequences have dissimilar correlation patterns, and correlations between individual *Legionella* ZOTUs are rare. This suggests the possibility that *Legionella* species live within different and defined ecological niches in biofilms, and that they interact/associate with different microorganisms (Rottjers and Faust 2018). This is, from an ecological point of view, an important aspect as more than 25 species within the genus *Legionella* are considered pathogenic and we believe that studying the ecology of non-pneumophila species is as important.

However, these observations should be read in the light of an unequal distribution of *Legionella* across biofilm samples. The approximate *Legionella* abundances ranged between 1E+3 and 1E+7 GU/hose, suggesting considerable but variable colonisation of the hoses. Moreover, the distribution of the ZOTUs across samples was also uneven, with sequences present in 2 to 81 biofilm samples (Fig. S4). Hence, it cannot be excluded that stochastic effects influenced which *Legionella* ZOTUs colonised specific samples.

### Critical considerations of the methods used to detect associations

The associations identified in this study display similarities, but also differences with other studies in the field (discussed above). This is influenced by a number of factors, which are not always clearly highlighted in similar studies:

(1) *Legionella quantification*: *Legionella* relative abundance derived from 16S rRNA ddPCR and *Legionella*-specific ddPCR data did not correlate particularly well with relative abundance derived from 16S amplicon sequencing data (Fig. 3B). While both carry valuable information, the two approaches have intrinsic differences; amplicon sequencing can be used as a quantitative measure, but it is always relative to the total reads. Amplification through ddPCR, on the other hand, allows absolute quantification of a specific target expressed in gene copies/µL. In an ecological context, a combination of both approaches is probably the best way to get as many information as possible and carry associations-based-analysis. Moreover, both these methods would amplify and detect non-viable *Legionella*, of which the importance in ecological-association studies is questionable. Previous studies with a similar focus used cultivation-based quantification methods (Ma et al. 2020, Scaturro et al. 2022), which also has known bias towards *L. pneumophila* and is limited by only analysing cells that grow on agar plates (Toplitsch et al. 2021). Clearly, the method of *Legionella* quantification would considerably affect the outcome of any correlation/association analysis.
(2) *Tools used to determine associations*: Changing the threshold on the relative abundance of *Legionella* (high *Legionella* vs low *Legionella*) affected the outcome of the Random Forest models (Fig. S6). This is partially due to the OOB errors of the models, which are especially higher for the prediction of low *Legionella* abundance. From a biological point of view, this is an important consideration: choosing the median relative abundance value as the threshold is statistically stronger as all the samples are part of the Random Forest analysis. Focusing only on the samples at the extremes would, however, in theory define more relevant predictors of the presence/absence of *Legionella*.

In addition, different statistical tools (i.e. SparCC and Random Forest analysis) predictably did not produce the same result but have complementary value. The Random Forest analysis identifies general predictors of the abundance of *Legionella* spp. and *L. pneumophila* in a given environment; SparCC analysis provides us with evidence on the separate ecological niches that different *Legionella* species occupy. In the former, the cause for high OOB values indicates the model is less accurate than desired while the latter highlights that associations of microorganisms ideally need to be resolved at a smaller taxonomic and spatial scale. Other studies have used linear regression or LefSe analysis to link *Legionella* to other microorganisms (Paranjape et al. 2020, Paranjape et al. 2020). Our methodology choice can be justified by the data collected in our study: targeted ddPCR allows us in fact to use the relative abundance of *Legionella* as an external variable that can be predicted by the amplicon sequencing results (validated moreover by the fact that *Legionella* is displayed as a predictor of high *Legionella* abundance). While not advocating for certain methods over others, it is obvious that the choice of method would affect the result. An ideal situation would be standardizing methods and protocols across different studies, and reporting as much metadata as possible, as to gain generalizable and comparable insights into *Legionella* ecology and microbial associations in diverse environments.

(3) *Biofilm vs water phase:* In our study, the biofilm phase was specifically collected and analysed, compared to other investigations where the water phase was analysed (Ma et al. 2020, Paranjape et al. 2020, Scaturro et al. 2022). While noting the challenges in biofilm sampling, we do believe that this choice is critical for the determination of associations between microorganisms. The microbial composition of biofilm and water is different and not necessarily reflective of each other (Chan et al. 2019), and therefore associations detected in the two phases are likely different. Also, *Legionella* are clearly recognised as biofilm-associated bacteria (Declerck 2010), hence, associations within this spatial niche should be most relevant. Finally, the bacterial composition of established biofilms bacterial composition is more stable in time, while water is a more dynamic system (Inkinen et al. 2014).

### Function vs taxonomy

A strong limitation of conventional amplicon sequencing is that only taxonomic information is obtained after processing, and it is often not possible to resolve the dataset at species level. This does not allow a good overview of the species dynamics in given samples, while making it also nearly impossible to look at specific functions shaping the ecology of the environments (Lajoie and Kembel 2019). Shotgun metagenomics may be one alternative approach (Eisen 2007, Perez-Cobas et al. 2020). The advantages of such a method compared to amplicon sequencing are a finer taxonomic resolution (it is possible in some cases to reconstruct entire genomes) and, more importantly, the detection of genes with their abundances) associated with specific functions. This would benefit investigations on *Legionella* ecology and interactions with other organisms as well. Our study identified several organisms correlating with/predicting the abundance of *Legionella* and we explored potentially explanations for these interactions. Using shotgun metagenomics in these studies would, inform on the specific functions associated with the abundance of *Legionella*, which would in turn help creating a more detailed overview on what characterizes an environment as favourable or undesirable for *Legionella* proliferation, being moreover able to link the genes to the organisms. We previously proposed potential probiotics approaches against *Legionella* in engineered aquatic ecosystems (Cavallaro et al. 2022), and reviewed studies that identified antagonistic behaviours of certain microorganisms. The use of shotgun metagenomics in observational studies can be a useful resource in this sense, as the identification of specific functions that make the environment undesirable for *Legionella* can be searched for in specific microorganisms to use in such an approach.

## Conclusions

- Non-chlorinated shower hose biofilms from the same building plumbing system are inhabited by diverse bacterial and eukaryotic communities, including widely varying relative abundances of *Legionella*.
- Eukaryotic communities are important for a complete understanding of the ecology of *Legionella* in plumbing systems, but a considerable expansion of 18S databases is crucial for the identification of key organisms and associations.
- A combination of bacterial and eukaryotic organisms statistically predicted the abundance of *Legionella* spp. (and *L. pneumophila*), but the direct ecological meaning of specific associations is ambiguous. The low predicting quality of the models, however, needs to be considered when putting the associations in a biological context.
- Analysing the associations looking at single amplicon sequence variants (rather than genus) gives an overview of potential interactions at species level, and a deeper ecological understanding of *Legionella* species dynamics.
- Moving towards function-based approaches (i.e. metagenomics of drinking water biofilms) will provide a better understanding of the mechanisms that affect *Legionella* abundance. This will in turn allow the use of specific known functions as predictors of *Legionella* abundance.

## Supplementary Material

xtad016_Supplemental_Files

## Data Availability

The data for this study have been deposited in the European Nucleotide Archive (ENA) at EMBL-EBI under accession number PRJEB61788.

## References

[bib1] Amaro F , Wang W, Gilbert JA et al. Diverse protist grazers select for virulence-related traits in Legionella. ISME J. 2015;9:1607–18. 25575308 10.1038/ismej.2014.248 PMC4478701

[bib2] Bairey E , Kelsic ED, Kishony R. High-order species interactions shape ecosystem diversity. Nat Commun. 2016;7:12285. 27481625 10.1038/ncomms12285 PMC4974637

[bib3] Bauer M , Mathieu L, Deloge-Abarkan M et al. Legionella bacteria in shower aerosols increase the risk of Pontiac fever among older people in retirement homes. J Epidemiol Community Health. 2008;62:913–20. 18791050 10.1136/jech.2007.063784

[bib4] Benitez AJ , Winchell JM. Clinical application of a multiplex real-time PCR assay for simultaneous detection of Legionella species, Legionella pneumophila, and Legionella pneumophila serogroup 1. J Clin Microbiol. 2013;51:348–51. 23135949 10.1128/JCM.02510-12 PMC3536254

[bib5] Berendt RF . Influence of Blue Algae (Cyanobacteria) on survival of Legionella pneumophila in aerosols. Infect Immun. 1981;32:690–2. 6788701 10.1128/iai.32.2.690-692.1981 PMC351502

[bib6] Boamah DK , Zhou G, Ensminger AW et al. From many hosts, one accidental pathogen: the diverse protozoan hosts of Legionella. Front Cell Infect Microbiol. 2017;7:477. 29250488 10.3389/fcimb.2017.00477 PMC5714891

[bib7] Buse HY , Ashbolt NJ. Differential growth of Legionella pneumophila strains within a range of amoebae at various temperatures associated with in-premise plumbing. Lett Appl Microbiol. 2011;53:217–24. 21623847 10.1111/j.1472-765X.2011.03094.x

[bib8] Caporaso JG , Lauber CL, Walters WA et al. Global patterns of 16S rRNA diversity at a depth of millions of sequences per sample. Proc Natl Acad Sci USA. 2011;108 Suppl 1:4516–22. 20534432 10.1073/pnas.1000080107 PMC3063599

[bib9] Carr A , Diener C, Baliga NS et al. Use and abuse of correlation analyses in microbial ecology. ISME J. 2019;13:2647–55. 31253856 10.1038/s41396-019-0459-z PMC6794304

[bib10] Cavallaro A , Rhoads WJ, Huwiler SG et al. Potential probiotic approaches to control Legionella in engineered aquatic ecosystems. FEMS Microbiol Ecol. 2022;98. 10.1093/femsec/fiac071 PMC9333994 35679082

[bib11] Chambers ST , Slow S, Scott-Thomas A et al. Legionellosis caused by non-Legionella pneumophila species, with a focus on Legionella longbeachae. Microorganisms. 2021;9:291. 33572638 10.3390/microorganisms9020291 PMC7910863

[bib12] Chan S , Pullerits K, Keucken A et al. Bacterial release from pipe biofilm in a full-scale drinking water distribution system. NPJ Biofilms Microbiomes. 2019;5:9. 30820334 10.1038/s41522-019-0082-9 PMC6385293

[bib13] Chien M , Morozova I, Shi S et al. The Genomic sequence of the accidental pathogen Legionella pneumophila. Science. 2004;305:1966–8. 15448271 10.1126/science.1099776

[bib14] Chong J , Liu P, Zhou G et al. Using MicrobiomeAnalyst for comprehensive statistical, functional, and meta-analysis of microbiome data. Nat Protoc. 2020;15:799–821. 31942082 10.1038/s41596-019-0264-1

[bib15] Corre MH , Delafont V, Legrand A et al. Exploiting the richness of environmental waterborne bacterial species to find natural Legionella pneumophila competitors. Front Microbiol. 2018;9:3360. 30697209 10.3389/fmicb.2018.03360 PMC6340971

[bib16] Declerck P . Biofilms: the environmental playground of Legionella pneumophila. Environ Microbiol. 2010;12:557–66. 19678829 10.1111/j.1462-2920.2009.02025.x

[bib17] Delafont V , Mougari F, Cambau E et al. First evidence of amoebae-mycobacteria association in drinking water network. Environ Sci Technol. 2014;48:11872–82. 25247827 10.1021/es5036255

[bib18] Dey R , Bodennec J, Mameri MO et al. Free-living freshwater amoebae differ in their susceptibility to the pathogenic bacterium Legionella pneumophila. FEMS Microbiol Lett. 2009;290:10–17. 19016880 10.1111/j.1574-6968.2008.01387.x

[bib19] D'Souza G , Shitut S, Preussger D et al. Ecology and evolution of metabolic cross-feeding interactions in bacteria. Nat Prod Rep. 2018;35:455–88. 29799048 10.1039/c8np00009c

[bib20] Edgar RC , Flyvbjerg H. Error filtering, pair assembly and error correction for next-generation sequencing reads. Bioinformatics. 2015;31:3476–82. 26139637 10.1093/bioinformatics/btv401

[bib21] Eisen JA . Environmental shotgun sequencing: its potential and challenges for studying the hidden world of microbes. PLoS Biol. 2007;5:e82. 17355177 10.1371/journal.pbio.0050082 PMC1821061

[bib22] Falkinham JO 3rd , Hilborn ED, Arduino MJ et al. Epidemiology and ecology of opportunistic premise plumbing pathogens: legionella pneumophila, mycobacterium avium, and Pseudomonas aeruginosa. Environ Health Perspect. 2015;123:749–58. 25793551 10.1289/ehp.1408692 PMC4529011

[bib23] Fields BS , Benson RF, Besser RE. Legionella and Legionnaires' disease: 25 years of investigation. Clin Microbiol Rev. 2002;15:506–26. 12097254 10.1128/CMR.15.3.506-526.2002 PMC118082

[bib24] Gabrielli M , Dai Z, Delafont V et al. Identifying eukaryotes and factors influencing their biogeography in drinking water metagenomes. Environ Sci Technol. 2023;57:3645–60. 36827617 10.1021/acs.est.2c09010 PMC9996835

[bib25] Gebert MJ , Delgado-Baquerizo M, Oliverio AM et al. Ecological analyses of mycobacteria in showerhead biofilms and their relevance to Human health. Mbio. 2018;9:e01614–18. 30377276 10.1128/mBio.01614-18 PMC6212831

[bib26] Gleason JA , Newby R, Gaynor JJ et al. Legionella monitoring results by water quality characteristics in a large public water system. Environ Sci Pollut Res Int. 2023;30:55974–88., 36913019 10.1007/s11356-023-26198-9

[bib27] Granato ET , Meiller-Legrand TA, Foster KR. The evolution and ecology of bacterial warfare. Curr Biol. 2019;29:R521–37. 31163166 10.1016/j.cub.2019.04.024

[bib28] Guerrieri E , Bondi M, Sabia C et al. Effect of bacterial interference on biofilm development by Legionella pneumophila. Curr Microbiol. 2008;57:532–6. 18769851 10.1007/s00284-008-9237-2

[bib29] Guillou L , Bachar D, Audic S et al. The Protist Ribosomal Reference database (PR2): a catalog of unicellular eukaryote small sub-unit rRNA sequences with curated taxonomy. Nucleic Acids Res. 2013;41:D597–604. 23193267 10.1093/nar/gks1160 PMC3531120

[bib30] Hines SA , Chappie DJ, Lordo RA et al. Assessment of relative potential for Legionella species or surrogates inhalation exposure from common water uses. Water Res. 2014;56:203–13. 24681377 10.1016/j.watres.2014.02.013

[bib31] Ho J , Adeolu M, Khadka B et al. Identification of distinctive molecular traits that are characteristic of the phylum “Deinococcus-Thermus” and distinguish its main constituent groups. Syst Appl Microbiol. 2016;39:453–63. 27506333 10.1016/j.syapm.2016.07.003

[bib32] Inkinen J , Jayaprakash B, Siponen S et al. Active eukaryotes in drinking water distribution systems of ground and surface waterworks. Microbiome. 2019;7:99. 31269979 10.1186/s40168-019-0715-5 PMC6610866

[bib33] Inkinen J , Kaunisto T, Pursiainen A et al. Drinking water quality and formation of biofilms in an office building during its first year of operation, a full scale study. Water Res. 2014;49:83–91. 24317021 10.1016/j.watres.2013.11.013

[bib34] Ji P , Rhoads WJ, Edwards MA et al. Impact of water heater temperature setting and water use frequency on the building plumbing microbiome. ISME J. 2017;11:1318–30. 28282040 10.1038/ismej.2017.14 PMC5437349

[bib35] Kehe J , Ortiz A, Kulesa A et al. Positive interactions are common among culturable bacteria. Sci Adv. 2021;7:eabi7159. 34739314 10.1126/sciadv.abi7159 PMC8570599

[bib36] Lajoie G , Kembel SW. Making the most of trait-based approaches for microbial ecology. Trends Microbiol. 2019;27:814–23. 31296406 10.1016/j.tim.2019.06.003

[bib37] Lee KC , Dunfield PF, Morgan XC et al. Chthonomonas calidirosea gen. nov., sp. nov., an aerobic, pigmented, thermophilic micro-organism of a novel bacterial class, Chthonomonadetes classis nov., of the newly described phylum Armatimonadetes originally designated candidate division OP10. Int J Syst Evol Microbiol. 2011;61:2482–90. 21097641 10.1099/ijs.0.027235-0

[bib38] Leoni E , Legnami PP, Bucci Sabattini MA et al. Prevalence of Legionella spp. In swimming pool environment. Water Res. 2001;15:3749–53. 10.1016/s0043-1354(01)00075-6 11561639

[bib39] Lesnik R , Brettar I, Hofle MG. Legionella species diversity and dynamics from surface reservoir to tap water: from cold adaptation to thermophily. ISME J. 2016;10:1064–80. 26528838 10.1038/ismej.2015.199 PMC5029207

[bib40] Liu G , Bakker GL, Li S et al. Pyrosequencing reveals bacterial communities in unchlorinated drinking water distribution system: an integral study of bulk water, suspended solids, loose deposits, and pipe wall biofilm. Environ Sci Technol. 2014;48:5467–76. 24766451 10.1021/es5009467

[bib41] Logan-Jackson AR , Rose JB. Water age effects on the occurrence and concentration of Legionella species in the distribution system, premise plumbing, and the cooling towers. Microorganisms. 2021;10:81. 35056530 10.3390/microorganisms10010081 PMC8778510

[bib42] Ma AT , Daniels EF, Gulizia N et al. Isolation of diverse amoebal grazers of freshwater cyanobacteria for the development of model systems to study predator–prey interactions. Algal Research. 2016;13:85–93.

[bib43] Ma X , Pierre D, Bibby K et al. Bacterial community structure correlates with Legionella pneumophila colonization of New York City high rise building premises plumbing systems. Environ Sci: Water Res Technol. 2020;6:1324–35.

[bib44] McMurdie PJ , Holmes S. phyloseq: an R package for reproducible interactive analysis and graphics of microbiome census data. PLoS One. 2013;8:e61217. 23630581 10.1371/journal.pone.0061217 PMC3632530

[bib45] McMurdie PJ , Holmes S. Waste not, want not: why rarefying microbiome data is inadmissible. PLoS Comput Biol. 2014;10:e1003531. 24699258 10.1371/journal.pcbi.1003531 PMC3974642

[bib46] Mondino S , Schmidt S, Rolando M et al. Legionnaires' Disease: state of the art knowledge of pathogenesis mechanisms of Legionella. Annu Rev Pathol. 2020;15:439–66. 31657966 10.1146/annurev-pathmechdis-012419-032742

[bib47] Muder RR , Victor LY. Infection due to Legionella species other than L. pneumophila. Clinical Infection Diseases. 2002;35:990–8. 10.1086/342884 12355387

[bib48] Mühlenberg W . Fatal travel-associated legionella infection caused by shower aerosols in a German hotel. Gesundheitswesen (Bundesverband Der Arzte Des Offentlichen Gesundheitsdienstes (Germany)). 1993;55:653–6. 8111164

[bib49] Neu L , Proctor CR, Walser JC et al. Small-scale heterogeneity in drinking water biofilms. Front Microbiol. 2019;10:2446. 31736893 10.3389/fmicb.2019.02446 PMC6828615

[bib50] Niculita-Hirzel H , Vanhove AS, Leclerc L et al. Risk exposure to Legionella pneumophila during showering: the difference between a classical and a water saving shower system. Int J Environ Res Public Health. 2022;19:3285. 35328980 10.3390/ijerph19063285 PMC8955837

[bib51] Paniagua AT , Paranjape K, Hu M et al. Impact of temperature on Legionella pneumophila, its protozoan host cells, and the microbial diversity of the biofilm community of a pilot cooling tower. Sci Total Environ. 2020;712:136131. 31931228 10.1016/j.scitotenv.2019.136131

[bib52] Paranjape K , Bedard E, Shetty D et al. Unravelling the importance of the eukaryotic and bacterial communities and their relationship with Legionella spp. Ecology in cooling towers: a complex network. Microbiome. 2020;8:157. 33183356 10.1186/s40168-020-00926-6 PMC7664032

[bib53] Paranjape K , Bedard E, Whyte LG et al. Presence of Legionella spp. In cooling towers: the role of microbial diversity, Pseudomonas, and continuous chlorine application. Water Res. 2020;169:115252. 31726393 10.1016/j.watres.2019.115252

[bib54] Pereira RP , Peplies J, Brettar I et al. Development of a genus-specific next generation sequencing approach for sensitive and quantitative determination of the Legionella microbiome in freshwater systems. BMC Microbiol. 2017;17:79. 28359254 10.1186/s12866-017-0987-5 PMC5374610

[bib55] Perez-Cobas AE , Gomez-Valero L, Buchrieser C. Metagenomic approaches in microbial ecology: an update on whole-genome and marker gene sequencing analyses. Microb Genom. 2020;6. 10.1099/mgen.0.000409 PMC7641418 32706331

[bib56] Proctor CR , Gächter M, Kötzsch S et al. Biofilms in shower hoses – choice of pipe material influences bacterial growth and communities. Environ Sci: Water Res Technol. 2016;2:670–82.

[bib57] Proctor CR , Reimann M, Vriens B et al. Biofilms in shower hoses. Water Res. 2018;131:274–86. 29304381 10.1016/j.watres.2017.12.027

[bib58] Reeder J , Huang M, Kaminker JS et al. MicrobiomeExplorer: an R package for the analysis and visualization of microbial communities. Bioinformatics. 2021;37:1317–8. 32960962 10.1093/bioinformatics/btaa838 PMC8193707

[bib59] Rhoads WJ , Sindelar M, Margot C et al. Variable Legionella response to building occupancy patterns and precautionary flushing. Microorganisms. 2022;10:555. 35336130 10.3390/microorganisms10030555 PMC8950775

[bib60] Rottjers L , Faust K. From hairballs to hypotheses-biological insights from microbial networks. FEMS Microbiol Rev. 2018;42:761–80. 30085090 10.1093/femsre/fuy030 PMC6199531

[bib61] Rowbotham TJ . Current views on the relationships between amoebae, legionellae and man. Isr J Med Sci. 1986;22:678–89. 3793451

[bib62] Sadiq FA , Hansen MF, Burmolle M et al. Trans-kingdom interactions in mixed biofilm communities. FEMS Microbiol Rev. 2022;46. 10.1093/femsre/fuac024 35640890

[bib63] Salinas MB , Fenoy S, Magnet A et al. Are pathogenic Legionella non-pneumophila a common bacteria in Water Distribution Networks?. Water Res. 2021;196:117013. 33813251 10.1016/j.watres.2021.117013

[bib64] Scaturro M , Chierico FD, Motro Y et al. Premise plumbing bacterial communities in four European cities and their association with Legionella. Frontiers in Microbiomes. 2023.2:10.3389/frmbi.2023.1170824 PMC12993568 41853366

[bib65] Sharma M , Khurana H, Singh DN et al. The genus Sphingopyxis: systematics, ecology, and bioremediation potential - A review. J Environ Manage. 2021;280:111744. 33280938 10.1016/j.jenvman.2020.111744

[bib66] Soler P , Moreno-Mesonero L, Zornoza A et al. Characterization of eukaryotic microbiome and associated bacteria communities in a drinking water treatment plant. Sci Total Environ. 2021;797:149070. 34303230 10.1016/j.scitotenv.2021.149070

[bib67] Stott MB , Lee KCY, Morgan XC et al. Chthonomonas. Bergey's Man System Archaea Bacteria. 2018; pp.1–6.

[bib68] Taylor M , Ross K, Bentham R. Legionella, protozoa, and biofilms: interactions within complex microbial systems. Microb Ecol. 2009;58:538–47. 19365668 10.1007/s00248-009-9514-z

[bib69] Tesh MJ , Miller RD. Amino Acid requirements for Legionella pneumophila growth. J Clin Microbiol. 1981;13:865–9. 6113250 10.1128/jcm.13.5.865-869.1981 PMC273905

[bib70] Tison DL , Pope DH, Cherry WB et al. Growth of Legionella pneumophila in association with blue-green algae (cyanobacteria). Appl Environ Microbiol. 1980;39:456–9. 6769388 10.1128/aem.39.2.456-459.1980 PMC291353

[bib71] Tobin JOH , Dunnill MS, French M et al. Legionnaires' disease in a traspant unit: isolation of the causative agent from showers bath. Lancet North Am Ed. 1980;2. 10.1016/s0140-6736(80)90005-7 6105294

[bib72] Toplitsch D , Platzer S, Zehner R et al. Comparison of updated methods for Legionella detection in environmental water samples. Int J Environ Res Public Health. 2021;18:5436. 34069615 10.3390/ijerph18105436 PMC8161308

[bib73] Toze S , Sly LI, MacRae IC et al. Inhibition of growth of Legionella species by heterotrophic plate count bacteria isolated from chlorinated drinking water. Curr Microbiol. 1990;21:139–43.

[bib74] Tsao HF , Scheikl U, Herbold C et al. The cooling tower water microbiota: seasonal dynamics and co-occurrence of bacterial and protist phylotypes. Water Res. 2019;159:464–79. 31128471 10.1016/j.watres.2019.04.028 PMC6554697

[bib75] Vosloo S , Sevillano M, Pinto A. Modified DNeasy PowerWater Kit® protocol for DNA extractions from drinking water samples. Protocolsio. 2019.

[bib76] Wadowsky RM , Yee RB. Satellite growth of Legionella pneumophila with an environmental isolate of Flavobacterium breve. Appl Environ Microbiol. 1983;46:1447–9. 6660882 10.1128/aem.46.6.1447-1449.1983 PMC239593

[bib77] Wilson DA , Reischl U, Hall GS et al. Use of partial 16S rRNA gene sequencing for identification of Legionella pneumophila and non-pneumophila Legionella spp. J Clin Microbiol. 2007;45:257–8. 17108074 10.1128/JCM.01552-06 PMC1828979

