# Supplementary material for: Legionella relative abundance in shower hose biofilms is associated with specific microbiome members
Source: FEMS Microbes. 2023 Aug 16;4:xtad016. doi: 10.1093/femsmc/xtad016 (PMC10496943; doi:10.1093/femsmc/xtad016)
Supplement: xtad016_Supplemental_Files [file xtad016_supplemental_files.zip › FEMSMC-2023-012 supplementary data - reviews and author response.docx]

**Reviews of manuscript FEMSMC-2023-012**

**Reviewer 1**

**Recommendation: Minor Revision**

The authors presented a relevant study on the ecology of Legionella in biofilms and its association with prokaryotic/eukaryotic organisms, highlighting protists that are key in Legionella biology. The methodology is accurate, combining ddPCR with marker gene sequencing, and the data is very well-explored (modelling, co-occurrence networks) and the results are nicely presented and discussed. Then, I only have some minor comments to improve the manuscript.

164-165: "Based on their concentration, samples were divided into three libraries, which were subsequently pooled together to a final concentration of 4 nM". This sentence is difficult to follow, typically, the sample concentration is adjusted, and then all pooled to 4nM, but you mentioned that libraries are pooling. Please clarify it.

Lines 214-217: I would remove section 3.1 Overview. It is not necessary to follow the paper.

Lines 233-234: Please refer to the genus name (without the X or X lineage) (check that in NCBI taxonomy names)

Line 243: Detail what H-values mean.

Lines 263-266: "No clear correlation was observed between the relative abundance of Legionella spp. and L. pneumophila. Importantly, the relative abundance of Legionella spp. calculated from the 16S rRNA amplicon sequencing and calculated with ddPCR correlated poorly (R2=0.18; Figure 3B)". I wonder if the 16S primers for the ddPCR are specific to Legionella or just the V4 region since, in that case, the whole microbiome will be amplified (Legionella and others). That could explain the low correlation between Legionella abundance and total biomass.

Line 358: What is the genus or the taxonomic level identified of the ZOTU193?

Lines 260-261: The ddPCR allows absolute quantification, while the authors refer to this estimate as relative abundance. Commonly relative abundance is used for the amplicon data when the number of reads of a taxon is divided by the total number of reads of the sample. I think it should be more precise that the authors use the ddPCR results for the models. Please, clarify or rephrase.

Line 436: "4.3. Some prokaryotic taxa are associated with Legionella abundance". Adding more specific discussion about those related taxa to Legionella could be interesting. For example, is there evidence in the literature of the association of those taxa with Legionella, or a possible explanation of the interaction mechanisms?

Line 557-558: "We used both ddPCR and 16S amplicon sequencing, and results from the two methods did not correlate particularly well (Figure 3B)". I think that, somehow, the two methods are not comparable since the sequencing is a quantitative or semi-quantitative approach but is always relative. At the same time, the ddPCR is a quantification method. Then it is necessary to use both to combine information, for example, by quantifying the total biomass with a 16S rRNA ddPCR and normalizing the sequencing data on these values. This paragraph could be better explained on this basis.

**Reviewer 2**

**Recommendation: Minor Revision**

This manuscript presents a study of the biofilm microbiota present in shower hoses, and its relationship with Legionella. Shower hoses are understudied, but most likely a very important source of LD. Importantly, the paper target biofilm in real system, which is another understudied facet of Legionella life cycle. Overall, the manuscripts make important contributions to the field.

The data collection and analysis seem sound and the manuscript is well written. I have minor comments pertaining to points that needs to be clarified.

General comments:

1. I appreciate that the authors discuss the pitfalls of the methods used in section 4.6. The authors indicated at line 175 that their 16S rDNA amplicon sequencing data was of very poor quality for the reverse reads, and only used the forward reads for their analysis. It would be important to comment on the reliability of their analysis using only the forward read. Other studies do this, so I don’t think this is a major issue, but could this have affected the downstream analysis? Could this have resulted in a high level of unassigned reads (line 223-224). How does these high values of unassigned reads compare with other study?

2. Additional details are required for the amplicon sequencing analysis (section 2.6). Please indicate how rarefaction was conducted and to what level. Was the default setting used for all the packages mentioned in that section?

3. Section 3.3 described the ddPCR data for Lspp and Lp. The authors present the data as the relative abundance against total 16S rRNA. This does not allow the reader to understand the abundance of Lp in those shower hoses. The level of colonization (GU per L or, in this case, maybe per hose) of the shower hoses by legionella is important for the discussion of the results, since this is the parameter that is used for surveillance. In other words, are we talking about severe colonization by Legionella, or "clean" hoses here? The authors could present their ddPCR data as the number of GU per hoses and used this value to carry association analysis with the microbiota (e.g. do the hoses most contaminated with Lspp or Lp have different microbiota). Also, the authors could show the absolute quantification of 16S rRNA by ddPCR as an indication of the amount of biofilm in each hose. This parameter could also be used to identify association between level of biofilm and the microbiota present.

4. In addition, if Lspp and Lp are present in minutes quantity (a few GU per hoses), then the observation that specific Legionella ZOTUs are associated with specific microbiota members is most likely due to the founder effect (one can imagine that due to stochastic effect ZOTU 193 colonized hose #3, but ZOTU 1176 colonized hose #10). The conclusions of the author that “separate ecological niches occupied by different Legionella species” (e.g. line 35) should be discussed in light of this possibility.

5. The authors mentioned several times that the shower hoses were "full of diversity" (e.g. line 432). I am not sure what data support this statement. The NMDS showed that the hoses are quite similar in terms of microbiota.

6. Section 4.3 discuss contradictory data between taxon associated with Legionella in different study. This is likely to due to difference in strains, which the authors mentioned lien 530. I would suggest moving this to section 4.3.

7. The authors used the term prokaryotes. I would recommend avoiding this term as Bacteria and Archaea are considered distinct domain and that Archaea does not seem to have been studied in this paper.

8. it would be useful for the readers to know more about shower hoses as sources of LD. Maybe add some epidemiological data in the introduction, if at all available in the literature. I presume that several nosocomial cases or sporadic cases have been associated with shower hoses.

9. More information about the building plumbing systems could be added, if available: material of the water distribution system, temperature, at which it is maintained, whether the temperature is mitigated at the hose or at a distant location, type of water heater, age of the building, etc.

Specific comments.

Figure 1: clarify how the unassigned ASVs are displayed in the figure.

Figure 4 and 5: fonts is very small, probably too small to read in printed copies.

Supplementary Figure 3: what does the white bar means?

Figure 6: “in the top right” do the authors mean “far left”?

Line 94: I would suggest citing the following article here, that studied biofilm in a pilot system: Paniagua, A. T., Paranjape, K., Hu, M., Bédard, E. & Faucher, S. P. Impact of temperature on Legionella pneumophila, its protozoan host cells, and the microbial diversity of the biofilm community of a pilot cooling tower. Science of the Total Environment, The 712, 136131 (2020).

Line 414: I would suggest citing: Pereira, R. P. A., Peplies, J., Brettar, I. & Höfle, M. G. Development of a genus-specific next generation sequencing approach for sensitive and quantitative determination of the Legionella microbiome in freshwater systems. Bmc Microbiol 17, 1–14 (2017), here. They were the first to described diversity of Legionella within a system.

**Author's Response to Decision Letter for (FEMSMC-2023-012)**

We herewith resubmit the revised version of our manuscript entitled “Legionella relative abundance in shower hose biofilms is associated with specific microbiome members”, as well as the detailed responses to the reviewer questions and comments (below).

We express our gratitude for the insightful and constructive comments of both reviewers. We addressed all their comments and incorporated changes to the text where applicable. We have uploaded a clean version and a version with track changes, while the supplementary information contain one new image in response to a question from reviewer 2. We are satisfied that the changes improved the overall quality of the manuscript, and hope that it would justify acceptance of this work.

On behalf of all the authors

Frederik Hammes

RESPONSE TO REVIEWERS for

Legionella relative abundance in shower hose biofilms is associated with specific microbiome members

Alessio Cavallaro, William J. Rhoads, Émile Sylvestre, Thierry Marti, Jean-Claude Walser, Frederik Hammes

REVIEWER 1

AUTHORS: The comprehensive and constructive comments of the reviewer are much appreciated and strengthened/clarified several sections of the manuscript. Changes/additions to the revised manuscript are indicated with the appropriate page/line numbers.

1. 164-165: "Based on their concentration, samples were divided into three libraries, which were subsequently pooled together to a final concentration of 4 nM". This sentence is difficult to follow, typically, the sample concentration is adjusted, and then all pooled to 4nM, but you mentioned that libraries are pooling. Please clarify it.

AUTHORS: Done as suggested. The original text described an extra step that we took in order to facilitate the laboratory procedure, given that samples had different ranges of concentration. Specifically, the samples were pooled together based on similarity in concentration, then adjusted and then pooled all together to 4nM. For clarity, the revised text does not contain this extra step.

Revised text: L171-172 “Sample concentration was adjusted and samples were subsequently pooled together in a library at a concentration of 4nM.”

2. Lines 214-217: I would remove section 3.1 Overview. It is not necessary to follow the paper.

AUTHORS: Done as suggested.

3. Lines 233-234: Please refer to the genus name (without the X or X lineage) (check that in NCBI taxonomy names).

AUTHORS: Done as suggested.

4. Line 243: Detail what H-values mean.

AUTHORS: Done as suggested.

Revised text: L247 “Alpha-diversity was measured using the Shannon index (H), which evaluates both richness and evenness of the species present. The analysis for the 16S dataset shows high H values within samples, ranging between 3 and 5, with 11 samples having less than 3. The same analysis performed on the 18S dataset shows that most samples range between 3 and 4.8, while 12 samples have values below 3 (“H”, Supplementary Figure 2).”

5. Lines 263-266: "No clear correlation was observed between the relative abundance of Legionella spp. and L. pneumophila. Importantly, the relative abundance of Legionella spp. calculated from the 16S rRNA amplicon sequencing and calculated with ddPCR correlated poorly (R2=0.18; Figure 3B)". I wonder if the 16S primers for the ddPCR are specific to Legionella or just the V4 region since, in that case, the whole microbiome will be amplified (Legionella and others). That could explain the low correlation between Legionella abundance and total biomass.

AUTHORS: In this study, in both cases (sequencing and 16S ddPCR) the primers used targeted the V4 region of the 16S rRNA gene. The two relative abundances discussed in the extract are the one calculated using the 16S amplicon sequencing data and the ddPCR data normalized against the total 16S rRNA gene. The text has been modified for clarity as follows:

Revised text: L271 “Importantly, the relative abundance of Legionella spp. calculated from the 16S rRNA amplicon sequencing data and that calculated with ddPCR (Legionella spp. and L. pneumophila relative to the total 16S rRNA) correlated poorly (R2=0.18; Figure 3B). Unless stated otherwise, the following analysis uses the targeted ddPCR data.

6. Line 358: What is the genus or the taxonomic level identified of the ZOTU193?

AUTHORS: Modified for clarity.

Revised text: L369 “For example, ZOTU193 was the most prevalent sequence assigned to the genus Legionella in the dataset (Supplementary Figure 3) and was also the one displaying the most correlations (67), the majority of which were positive.”

7. Lines 260-261: The ddPCR allows absolute quantification, while the authors refer to this estimate as relative abundance. Commonly relative abundance is used for the amplicon data when the number of reads of a taxon is divided by the total number of reads of the sample. I think it should be more precise that the authors use the ddPCR results for the models. Please, clarify or rephrase.

AUTHORS: We clarified this further. In this study, the absolute quantification given by ddPCR was normalized against the total 16S rRNA in order to get a relative abundance measure (to allow for comparison/integration with sequencing data, etc.). The text has been modified for clarity.

Revised text: L265 “We performed ddPCR for specific quantification of Legionella spp. and L. pneumophila, as well as for quantification of total 16S rRNA gene abundance (Figure 3). Subsequently, the abundance of Legionella spp. and L. pneumophila was calculated relative to the total 16S rRNA abundance, and this relative abundance was compared across samples.

8. Line 436: "4.3. Some prokaryotic taxa are associated with Legionella abundance". Adding more specific discussion about those related taxa to Legionella could be interesting. For example, is there evidence in the literature of the association of those taxa with Legionella, or a possible explanation of the interaction mechanisms?

AUTHORS: We modified the text adding some additional information regarding the genera detected in this study.

Revised text: L474 “Any specific associations between the genera detected in this study and Legionella were, to our knowledge, not previously described. Notably, two genera among the ones associated with Legionella (i.e., Meiothermus and Chthonomonas) are thermophilic (Lee et al., 2011, Ho et al., 2016), suggesting the possibility that some of these associations is dictated by environmental preferences (i.e. shared niches) rather than actual direct interactions. However, Chthonomonas spp. is also known for establishing mutualistic relationship with other organisms, specifically in biofilms (Stott et al., 2018).”

9. Line 557-558: "We used both ddPCR and 16S amplicon sequencing, and results from the two methods did not correlate particularly well (Figure 3B)". I think that, somehow, the two methods are not comparable since the sequencing is a quantitative or semi-quantitative approach but is always relative. At the same time, the ddPCR is a quantification method. Then it is necessary to use both to combine information, for example, by quantifying the total biomass with a 16S rRNA ddPCR and normalizing the sequencing data on these values. This paragraph could be better explained on this basis.

AUTHORS: We indeed calculated relative abundance from the ddPCR data as well. While we acknowledge differences between the methods, this was necessary in order to do the analysis for L. pneumophila specifically (which cannot be resolved with 16S amplicon sequencing alone). We took the suggestion of the reviewer to modify the paragraph as follows:

Revised text: L574 “Legionella quantification: Legionella relative abundance derived from 16S rRNA ddPCR and Legionella-specific ddPCR data did not correlate particularly well with relative abundance derived from 16S amplicon sequencing data (Figure 3B). While both carry valuable information, the two approaches have intrinsic differences; amplicon sequencing can be used as a quantitative measure, but it is always relative to the total reads. Amplification through ddPCR, on the other hand, allows absolute quantification of a specific target expressed in gene copies/µL. In an ecological context, a combination of both approaches is probably the best way to get as much information as possible and carry associations-based-analysis.”

___________

REVIEWER 2

AUTHORS: The comprehensive and constructive comments of the reviewer are much appreciated and strengthened/clarified several sections of the manuscript. Changes/additions to the revised manuscript are indicated with the appropriate page/line numbers.

General comments:

1. I appreciate that the authors discuss the pitfalls of the methods used in section 4.6. The authors indicated at line 175 that their 16S rDNA amplicon sequencing data was of very poor quality for the reverse reads, and only used the forward reads for their analysis. It would be important to comment on the reliability of their analysis using only the forward read. Other studies do this, so I don’t think this is a major issue, but could this have affected the downstream analysis? Could this have resulted in a high level of unassigned reads (line 223-224)? How does these high values of unassigned reads compare with other study?

AUTHORS: In the particular case of our sequencing run, since the chosen primer pair only cover the V4 region, the missing reverse read does not affect the length of the amplicon, which is of the correct size. In this case, the reverse read would only help with error correction, which is anyway incorporated in the analysis when assigning ZOTUs. All the details regarding the pipeline executed can be found in the supplementary reports. The text has been adjusted for clarity as follows:

Revised text: L182 “For the 16S dataset, because of the low quality of the reverse read, only the forward read has been processed further and used for the rest of the analysis. Since the primers amplified only the V4 region of the 16S, excluding the reverse read from the analysis did not cause a loss in length of the amplicon.”

2. Additional details are required for the amplicon sequencing analysis (section 2.6). Please indicate how rarefaction was conducted and to what level. Was the default setting used for all the packages mentioned in that section?

AUTHORS: Details added as suggested.

Revised text 1: L192 “Samples were not rarified to avoid the loss of data due to differences in the sequencing depth (McMurdie & Holmes, 2014).”

Revised text 2: L199 “Unless otherwise specified, all packages were operated using the default settings.”

3. Section 3.3 described the ddPCR data for Lspp and Lp. The authors present the data as the relative abundance against total 16S rRNA. This does not allow the reader to understand the abundance of Lp in those shower hoses. The level of colonization (GU per L or, in this case, maybe per hose) of the shower hoses by legionella is important for the discussion of the results, since this is the parameter that is used for surveillance. In other words, are we talking about severe colonization by Legionella, or "clean" hoses here? The authors could present their ddPCR data as the number of GU per hoses and used this value to carry association analysis with the microbiota (e.g., do the hoses most contaminated with Lspp or Lp have different microbiota). Also, the authors could show the absolute quantification of 16S rRNA by ddPCR as an indication of the amount of biofilm in each hose. This parameter could also be used to identify association between level of biofilm and the microbiota present.

AUTHORS: We agree that absolute abundance data would be interesting and valuable. However, in our study we focussed on relative, rather than absolute, abundance, as from an ecological perspective it was important to understand how different organisms associated relative to one another. Unfortunately, the exact total biofilm volumes were not quantified during extraction/filtration, and hence a direct conversion to absolute abundance (e.g., Legionella/hose) is not shown in the manuscript. However, based on the ddPCR data and the average biofilm volumes filtered, Legionella spp. were present at values between 10^3 – 10^7 GU/hose, suggesting relatively high Legionella colonization in most samples.

Revised Text: L276 “This study focused on the interactions between microbiome members and the absolute abundance of Legionella in the hoses were not quantified. However, based on the ddPCR data and the average biofilm volumes sampled, Legionella spp. were present at values between approximately 1E+3 – 1E+7 GU/hose., which is in agreement with previous studies (Proctor et al., 2018). L. pneumophila were considerably less abundant at approximate values ranging from 0 to 1E+06 GU/hose.”

The rest of the revised text incorporates both comment 3 and 4, and is shown as a reply to comment 4.

4. In addition, if Lspp and Lp are present in minutes quantity (a few GU per hoses), then the observation that specific Legionella ZOTUs are associated with specific microbiota members is most likely due to the founder effect (one can imagine that due to stochastic effect ZOTU 193 colonized hose #3, but ZOTU 1176 colonized hose #10). The conclusions of the author that “separate ecological niches occupied by different Legionella species” (e.g. line 35) should be discussed in light of this possibility.

AUTHORS: We believe this is a critical point and appreciate the reviewer’s comment. Although we do not show our data in the form of GU per hose, the estimation provided in reply to the previous comment suggests that Legionella was present in most hoses in high numbers, but in a broad range. To add to this, an additional analysis shows how the frequency of detection of the individual ZOTUs across samples ranges between 2 and 81. This information combined suggests that despite the high level of colonization of Legionella, stochastic effects due to the ranges of Legionella cannot be excluded, and the discussion has been adapted accordingly.

Revised Text: L563 “However, these observations should be read in the light of an unequal distribution of Legionella across biofilm samples. The approximate Legionella abundances ranged between 1E+3 and 1E+7 GU/hose, suggesting considerable but variable colonisation of the hoses. Moreover, the distribution of the ZOTUs across samples was also uneven, with sequences present in 2 to 81 biofilm samples (Supplementary Figure 4). Hence, it cannot be excluded that stochastic effects influenced which Legionella ZOTUs colonised specific samples.”

5. The authors mentioned several times that the shower hoses were "full of diversity" (e.g. line 432). I am not sure what data support this statement. The NMDS showed that the hoses are quite similar in terms of microbiota.

AUTHORS: The authors agree with the comment and modified for clarity, diversity will be referred to as “richness” to highlight the biofilms are home to many organisms, while the samples appear similar in terms of community structure.

6. Section 4.3 discuss contradictory data between taxon associated with Legionella in different study. This is likely to due to difference in strains, which the authors mentioned lien 530. I would suggest moving this to section 4.3.

AUTHORS: We recognize the importance of the point highlighted by the reviewer. In the manuscript we discussed the limitations of results presented at genus level (or higher) (section 4.3) as a potential factor explaining the contradictory data between taxa associated with Legionella.

L487: “For example, previous studies analysed exclusively the water phase, in contrast with our study that focused on the biofilm phase. Moreover, the taxonomic classification at the genus (sometimes class) level that was used in all of these studies does not allow for specific identification of the organisms involved, and different species within the same genus might exhibit different associations with the same organism. We explored the latter point by looking at the correlations that specific Legionella ZOTUs exhibit in a SparCC analysis (Figure 6; discussed below).”

7. The authors used the term prokaryotes. I would recommend avoiding this term as Bacteria and Archaea are considered distinct domain and that Archaea does not seem to have been studied in this paper.

AUTHORS: “Prokaryotes” and “prokaryotic” modified with “bacteria/bacterial” as suggested.

8. It would be useful for the readers to know more about shower hoses as sources of LD. Maybe add some epidemiological data in the introduction, if at all available in the literature. I presume that several nosocomial cases or sporadic cases have been associated with shower hoses.

AUTHORS: We agree with the comment and modified the text adding some information about shower hoses as a source of LD.

Revised text: L64 “Shower hoses have been previously linked to the transmission of Legionella through inhalation of water aerosol containing the bacteria (Niculita-Hirzel et al., 2022), and ranked second in a relative ranking of Legionella exposure pathways from common household water uses (Hines et al., 2014). In a few cases, showers have been directly linked to Legionellosis transmission, specifically in nursing homes, hospitals and hotels (Tobin et al., 1980, Mühlenberg, 1993, Bauer et al., 2008), although limited information regarding showers as route of transmission is overall available.”

9. More information about the building plumbing systems could be added, if available: material of the water distribution system, temperature, at which it is maintained, whether the temperature is mitigated at the hose or at a distant location, type of water heater, age of the building, etc.

AUTHORS: We fully appreciate the importance of such data. However, as stated in section 2.1, we could not provide any additional information on the specific plumbing system (e.g. use frequency, temperature, etc) due to privacy protection concerns.

Specific comments.

Figure 1: clarify how the unassigned ASVs are displayed in the figure.

AUTHORS: Caption modified for clarity:

Revised text: L219 “Nodes are shown until the last level that is taxonomically assigned. Unclassified taxa (not shown) at genus level account for 64.4±1.4% of the bacterial community and 85.3±1.2% of the eukaryotic community.”

Figure 4 and 5: fonts is very small, probably too small to read in printed copies.

AUTHORS: This may indeed be an issue. We had some trouble adjusting the font of the figure without losing information. If accepted, we will check the paper proof version and address again the problem if necessary.

Supplementary Figure 3: what does the white bar means?

AUTHORS: Caption was adapted for clarity.

Revised text: “A white bar indicates that no Legionella ZOTUs were detected in a particular sample.”

Figure 6: “in the top right” do the authors mean “far left”?

AUTHORS: Modified accordingly.

Line 94: I would suggest citing the following article here, that studied biofilm in a pilot system: Paniagua, A. T., Paranjape, K., Hu, M., Bédard, E. & Faucher, S. P. Impact of temperature on Legionella pneumophila, its protozoan host cells, and the microbial diversity of the biofilm community of a pilot cooling tower. Science of the Total Environment, The 712, 136131 (2020).

AUTHORS: Reference added as suggested.

Line 414: I would suggest citing: Pereira, R. P. A., Peplies, J., Brettar, I. & Höfle, M. G. Development of a genus-specific next generation sequencing approach for sensitive and quantitative determination of the Legionella microbiome in freshwater systems. Bmc Microbiol 17, 1–14 (2017), here. They were the first to described diversity of Legionella within a system.

AUTHORS: Reference added as suggested.

**Reviews of manuscript FEMSMC-2023-012.r1**

**Reviewer 1**

**Recommendation: Accept**

The modifications required to accept the article for publication have been made.

**Reviewer 2**

**Recommendation: Accept**

All of my comments were addressed in a satisfactory manner.

Minor correction: section 3 should be renumbered (L224 should be section 3.1).
